# Reduced Glutathione Mediates Pheno-Ultrastructure, Kinome and Transportome in Chromium-Induced *Brassica napus* L.

**DOI:** 10.3389/fpls.2017.02037

**Published:** 2017-12-11

**Authors:** Rafaqat A. Gill, Basharat Ali, Su Yang, Chaobo Tong, Faisal Islam, Muhammad Bilal Gill, Theodore M. Mwamba, Skhawat Ali, Bizeng Mao, Shengyi Liu, Weijun Zhou

**Affiliations:** ^1^Institute of Crop Science and Zhejiang Key Laboratory of Crop Germplasm, Zhejiang University, Hangzhou, China; ^2^Institute of Biotechnology, Zhejiang University, Hangzhou, China; ^3^Institute of Crop Science and Resource Conservation, University of Bonn, Bonn, Germany; ^4^Oil Crops Research Institute, Chinese Academy of Agricultural Sciences, Wuhan, China

**Keywords:** *Brassica napus* L., chromium, protein kinases, molecular transporters, reduced glutathione, transmission electron microscopy

## Abstract

Chromium (Cr) as a toxic metal is widely used for commercial purposes and its residues have become a potential environmental threat to both human and plant health. Oilseed rape (*Brassica napus* L.) is one of the candidate plants that can absorb the considerable quantity of toxic metals from the soil. Here, we used two cultivars of *B. napus* cvs. ZS 758 (metal-tolerant) and Zheda 622 (metal-susceptible) to investigate the phenological attributes, cell ultrastructure, protein kinases (PKs) and molecular transporters (MTs) under the combined treatments of Cr stress and reduced glutathione (GSH). Seeds of these cultivars were grown *in vitro* at different treatments i.e., 0, 400 μM Cr, and 400 μM Cr + 1 mM GSH in control growth chamber for 6 days. Results had confirmed that Cr significantly reduced the plant length, stem and root, and fresh biomass such as leaf, stem and root. Cr noticeably caused the damages in leaf mesophyll cells. Exogenous application of GSH significantly recovered both phenological and cell structural damages in two cultivars under Cr stress. For the PKs, transcriptomic data advocated that Cr stress alone significantly increased the gene expressions of *BnaA08g16610D, BnaCnng19320D*, and *BnaA08g00390D* over that seen in controls (Ck). These genes encoded both nucleic acid and transition metal ion binding proteins, and protein kinase activity (PKA) and phosphotransferase activities in both cultivars. Similarly, the presence of Cr revealed elite MT genes [*BnaA04g26560D, BnaA02g28130D*, and *BnaA02g01980D* (novel)] that were responsible for water transmembrane transporter activity. However, GSH in combination with Cr stress significantly up-regulated the genes for PKs [such as *BnaCnng69940D* (novel) and *BnaC08g49360D*] that were related to PKA, signal transduction, and oxidoreductase activities. For MTs, *BnaC01g29930D* and *BnaA07g14320D* were responsible for secondary active transmembrane transporter and protein transporter activities that were expressed more in GSH treatment than either Ck or Cr-treated cells. In general, it can be concluded that cultivar ZS 758 is more tolerant toward Cr-induced stress than Zheda 622.

## Introduction

At current development pace, pollution levels have significantly raised in biosphere (Swaminathan, 2003). Chromium (Cr), is a toxic metal that severely contaminates the soil, sediment, and ground water (Shanker et al., 2005). It is a non-essential metal; hence, there is no substantial evidence to support its role in plant metabolism. Thus, its transport system has not yet been elucidated. It is first absorbed by roots and then moves to the upper parts of the plant by a passive transport phenomenon (Skeffington et al., 1976; Zayed and Terry, 2003). However, only a few reports have stated that Cr uptake occurs through active transport mechanisms with the help of carriers such as that for sulfate. It is also difficult for iron, sulfur, and phosphorus carriers to transport and remove Cr ions (Shanker et al., 2005). Cr, after entering a plant body causes a reduction in plant growth, damages the young leaves, blocks the nutrient supply chain, produces wilting of the plant tops, and damages the roots (Sharma et al., 2003; Scoccianti et al., 2006).

Protein kinases (PKs) are documented as enzymatic-based regulators that modify other proteins by binding with serine/threonine or tyrosine via a chemically added phosphate group in a process called phosphorylation (Manning et al., 2002). This process usually changes the substrate protein's (target) cellular location, enzymatic activity, and association with other proteins. Moreover, kinases (such as histidine kinase) specifically phosphorylate histidine residues on target amino acids (Besant et al., 2003). The *Brassica napus* genome contains about 3695 PK-related transcripts that constituent about 3.4% of all *B. napus* related unigenes (for a total of 109,189) as in the present study. This ratio is larger than the human genome, which contains only 2% PKs (Manning et al., 2002). Furthermore, these PKs, including serine/threonine, mitogen-activated protein (MAP), and tyrosine specific PKs, are involved in modification of several proteins' activities and act as regulators of various cellular pathways, especially those related to signal transduction (Vlahopoulos and Zoumpourlis, 2004; Higashiyama et al., 2008).

In plants, transporters are responsible for channelizing mineral elements from one organ to another. Generally, a transport system in plants occurs *via* three methods: (1) uptake and discharge of water and solutes from single cells (such as the process of H_2_O absorption and minerals from the soil by root cells); (2) short distance movement of substances in adjacent cells such as sucrose loading from photosynthetic cells into phloem sieve tube cells; and (3) through long distance sap transport in the xylem and phloem vessels. Several minerals element-specific transporters have been previously studied, including a zinc (Zn)-mediated/iron (Fe)-standardized transporter (ZRT/IRT1), which is responsible for their respective transport in plants (Socha and Guerinot, 2014). The cell membrane adenosine triphosphatase (ATPase)-related type 1 transporters carry both calcium and manganese ions (Ca^2+^ and Mn^2+^, respectively) in the *Golgi* apparatus for the purpose of degradation and detoxification (Dürr et al., 1998).

Reduced glutathione (GSH) has been reported in many cell organelles, including the cytosol, chloroplasts, endoplasmic reticulum, vacuoles, and mitochondria. The chemical structure of GSH as a thiol group makes it suitable for enhancing the various enzymatic functions of many organisms. The unique nature of the thiol group helps to stabilize mercaptide–metal bonds. This type of stable bond formation in conjunction with higher water solubility presents GSH as a better scavenger against the toxicity of multiple metals (Gill et al., 2016a; Zlobin et al., 2017), reactive oxygen species (ROS) (Gill et al., 2014, 2015, 2016b), and other hazardous chemicals (Barrameda-Medina et al., 2017; Li et al., 2017). Previous reports have indicated that GSH plays a vital role in regulating the levels of oxygen singlet species (such as H_2_O_2_) that can be induced by heavy metal stress in plant cells (Shao et al., 2008; Gill et al., 2015). Furthermore, various researchers have also verified a role for GSH as an antioxidant in plant species under diverse capricious environmental conditions (Shao et al., 2008; Hasanuzzaman et al., 2017). Also, GSH activates plant scavenging mechanisms and conjugates with hazardous compounds. Thus, it plays a vital role in helping cells to alleviate various toxic elements induced stresses (Hasanuzzaman et al., 2017). The GSH binding mechanism is controlled by a gene called glutathione-S-transferase (Dixon et al., 2002). This gene also helps this conjugation progress from an infected (stressed) organelle to the vacuole. Accordingly, it can protect the cell from toxic effects (Klein et al., 2006; Yazaki, 2006).

Generally, GSH plays a role as the primary wall of protection against hazardous metals by inactivate metals before the induction of phyto-chelatins (PCs) reaches to an operational level. Because, GSH could be used as immediate substrate for the synthesis of PCs (Flores-Cáceres et al., 2015). Earlier studies have presented that biosynthesis of endogenous (intracellular) GSH and its accumulation can increase the tolerance of plants under the various unfavorable environmental conditions (Foyer and Noctor, 2011; Hasanuzzaman et al., 2017). In general, glutathione is oxidized to GSSG (oxidized glutathione) as part of its plant (inside cell) enzymatic defense. In order to maintain the level of GSH/GSSG in plant body, GR gene convert GSSG back to GSH by using NADPH (Foyer and Noctor, 2011; Seth et al., 2012). Recently, several studies have reported the role of exogenous GSH to enhance the plant growth and development and the antioxidant defense in various plants under different abiotic stress i.e. high temperature (Nahar et al., 2015a; Zhu et al., 2016), drought in mung bean (Nahar et al., 2015b), isoproturon in wheat (Alla and Hassan, 2014), Cd in rice and barley (Chen et al., 2010; Cai et al., 2011), Pb^+2^ in *Salvinia minima* (Estrella-Gómez et al., 2012), Cr in rice (Qiu et al., 2013), salt in tomato plants (Zhou et al., 2016, 2017), Cu and Zn in *B. napus* roots (Zlobin et al., 2017).

Recently, oilseed rape (*B. napus* L.) is well-known in bioremediation against heavy metals. Oilseed rape as a phytoremediator could serve as the source of a potentially profitable enterprise (Grispen et al., 2006). To date, literature studies have clarified that *B. napus* behaved differently under Cr-induced stress conditions as disclosed by physio-morphic, biochemical, and cell structural attributes (Gill et al., 2014, 2015, 2016a). Furthermore, our recent findings (Gill et al., 2016b) stated that GSH significantly up-regulated the genomic changes/gene expression that occurred under Cr toxicity in two *B. napus* cultivars. To our knowledge, there is no report that addresses the key issues of *B. napus* regarding the phenology, cell-ultrastructure, and particularly PKs, and MTs under Cr-toxicity and its alleviation through GSH application. Therefore, in the present study our aim was to explore the alleviating role of GSH regarding pheno-ultrastructure, kinome and transportome in Cr-induced *B. napus*.

## Materials and methods

### Plant material

Two cultivars of *B. napus* (ZS 758, black seeded and Zheda 622, yellow-seeded) were selected for this experiment. In our previous studies, we studied four cultivars i.e., ZS 758, Zheda 619, ZY 50, and Zheda 622 under Cr toxicity, and differentiated susceptible and resistance cultivars against Cr stress (Gill et al., 2014, 2015). These four cultivars are leading cultivars in the Yangtze River region, the main rapeseed production area in China. Therefore, in the present study we choose one tolerant cultivar (ZS 758) and one sensitive cultivar (Zheda 622). The mature seeds of these two cultivars were obtained from the College of Agriculture and Biotechnology, Zhejiang University, Hangzhou (China).

### Growth conditions

Fully grown seeds were washed with distilled H_2_O carefully. In a Petri dish, 60 seeds were positioned on a wet filter paper and incubated overnight. After germination, total 30 seedlings were selected randomly for each treatment and then transferred to a plastic box (12 cm^2^, having a sponge inside and a full Hoagland's nutrient solution). After 2 days of acclimatization, homogeneous seedlings were subjected to different combinations of Cr and GSH i.e., control (Ck), 400 μM Cr alone and 400 μM Cr + 1.0 mM GSH for 6 days. These treatments were repeated thrice and Cr concentration was selected by our previous work (Gill et al., 2014, 2015). The GSH levels were slected on the basis of our preliminary results in which we used 0, 0.5, 1, 1.5 and 2.0 mM concentrations along with Cr 400 μM. We found that 1.0 mM GSH level was best among all compared than Cr alone treatment. In our preliminary results, we also found that GSH level (1 mM) significantly improved plant growth under Cr stress as compared to other GSH levels (0.5, 2). Further, we also found that 1 mM GSH did not show any significant results as compared than control, therefore we did not use GSH alone treatment in the present experiment. The K_2_Cr_2_O_7_ salt was used to set up different Cr concentrations in the solution, and a Hoagland's solution (full-strength) was used as a fundamental medium with triplicates. The investigation was executeed in a chamber with a day/night temperatures of 24/16°C, respectively. A day length of 16-hr and irradiance of 300 μmol m^−2^ s^−1^ was used. The relative humidity was in the range of 60–70%. After 6 days, seedlings of both cultivars were harvested and data related to leaf fresh biomass were measured. Samples for TEM micrographs from leaf were taken immediately at the time of harvesting. For other parameters such as RNA-Seq and RT-PCR, fresh leaf samples were collected in the liquid nitrogen and then stored at −80°C until process them for further analysis.

### Phenological attributes

To measure the physiological parameters, plants were detached into leaves, stem, and roots. The physiological parameters were measured in the form of length of the whole plant, stem, root, and the leaf area. The fresh biomass of the rapeseed plants was quantified separately according to the previously described methodology of Zhang et al. (2008) and weighed immediately after turning off the experiment in the form of six plants per treatment by following the method of Momoh and Zhou (2001).

### Ultra-structural observations

Leaf fragments without veins (about 1 mm^2^) and root tips (about 2–3 mm) were collected from randomly selected plants for electron-microscopic study. In addition, further processes were completed according to Gill et al. (2014).

### Total RNA extraction, reliability assessment and RNA-sequence analyses

Total RNA was extracted manually from six samples of both cultivars with TRIZOLE-reagent (Invitrogen, USA). The integrity of samples was verified by minimum RNA integrated number of 8 by the 2100 bio-analyzer (Agilent). After, RNA samples were first treated with DNase I (Takara Biotechnology, China) to degrade any possible DNA contamination. Then the mRNA was enriched by using the oligo (dT) magnetic beads. The fragmentation buffer was added to fragment the mRNA to small sizes (~200 bp). After that, the first strand of cDNA was synthesized by using random hexamer primer. Buffer, dNTPs, RNase H, and DNA polymerase I added to synthesize the second strand. The double strand cDNA was purified with magnetic beads. End reparation and 3′-end single nucleotide A, adenine addition was then performed. Finally, the sequencing adaptors were ligated to the fragments. The fragments are enriched by PCR amplification. During the QC step, Agilent 2100 Bio-analyzer and ABI StepOnePlus Real-Time PCR System were used to qualify and quantify the sample library. The library products were ready for sequencing via Illumina HiSeq™ 2000 or other sequencer when necessary.

### Quantification of gene expression

The results of RNA-sequencer were cleaned from adapter sequences, mismatch and low-quality read with the help of internal software called filter_fq and then data saved as “.fastq” files. After that clean reads were mapped to reference sequences set using SOAPaligner/SOAP2 method that was previously described by Li et al. (2009), http://soap.genomics.org.cn/soapaligner.html. The expression level for each gene was calculated by using reads per kb per million reads (RPKM) method (Mortazavi et al., 2008).

### Screening of differentially expressed genes (DEGs), group differentially expressed genes

The differentially expressed genes (DEGs) were screened according to Audic and Claverie (1997) with some modifications. To determine the threshold of *P*-value in multiple samples, the FDR (False Discovery Rate) method was employed (Bajguz, 2010). Group differentially expressed genes were screened by the NOIseq method (Benjamini and Yekutieli, 2001).

### Estimation of protein kinases (PKS) and molecular transporters (MTs)

In order to sort out the PKs and MTs we had blasted the All-unigenes to non-redundant (nr) database. After that, we separated these PKs and MTs by using R software (https://www.r-project.org/) on the basis of *E*-value that ranges from 10 to 5.

### RNA extraction, cDNA synthesis, and quantitative real-time PCR (qRT-PCR) assays

Total RNA of six samples was extracted from 0.2 g of fresh leaf tissues of two cultivars by using the TRIZOLE-reagent (manual) (Sangon, China). The HiScript® II Q RT SuperMix for qPCR with gDNA wiper kit (Vazyme Biotech Co., Ltd.) was used for cDNA synthesis. The all six cDNA samples were assayed by qRT-PCR in the LightCycler® 96 System (Roche, Switzerland) using the AceQ® qPCR SYBR Green Master Mix (Vazyme Biotech Co., Ltd.). The reaction consisted of denaturation at 95°C for 30 s, followed by 40 cycles of denaturation at 95°C for 10 s, annealing at 60°C for 30 s and finally in dissociation process, denatured at 95°C for 15 s, annealing at 60°C for 60 s and denaturation at 95°C for 15 s. The primers for the corresponding elite transcripts regarding PKs and MTs were designed based on mRNA (*B. napus*) and are presented in the Table S1. Here, Brassica actin gene was used as an internal control. Moreover, we followed the earlier described quantification method of Livak and Schmittgen (2001).

### Statistical approaches

The significance of differences between black and yellow seeded *B. napus* cultivars in physiological, RNA-Seq and RT-PCR data were examined. The experiment was carried out through a randomized design. The results are the mean ± SD of at least three independent replicates and were analyzed using data processing system (DPS) statistical software package. We used two way ANOVA and then followed by the Duncan's Multiple Range Test (DMRT) (Tang and Zhang, 2013). The difference at *P*, 0.05 and 0.01 is considered as significant and highly significant, respectively.

## Results

### Phenological parameters

Results related to physiological attributes such as stem and root lengths and fresh biomass of different plant organs are presented in Table 1. Data showed that 400 μM Cr reduced stem length by 16% in ZS 758 and 27% in Zheda 622. Cr, at the same concentration, reduced the root length to 12% in ZS 758 and 35% in Zheda 622 when compared with the untreated control (Ck). However, exogenous application of GSH enhanced the stem length by 8% in ZS 758 and 14% in Zheda 622. In the same way, GSH added to 400 μM Cr increased root length by 12% in ZS 758 and 13% in Zheda 622. In addition, fresh leaf weight was reduced by 33% in ZS 758 and 50% in Zheda 622. Fresh stem weight was decreased by 38% in ZS 758 and 53% in Zheda 622. In the case of the roots, fresh biomass was reduced by 34% in ZS 758 and 53% in Zheda 622 under Cr-induced toxicity when compared with control. Exogenously applied GSH enhanced fresh biomass ingredients; leaf weight was increased by 25%, stem by 33%, and root by 30% in ZS 758. Similarly, GSH increased the leaf biomass contents of Zheda 622 by 36%, stem by 47%, and root by 57% when compared with treated plants (400 μM Cr). Interestingly, our results highlighted that ZS 758 proved more tolerant against Cr stress than Zheda 622. Moreover, GSH-enhanced percentages were more noticeable in Zheda 622 under stress conditions.

**Table 1:** Analyses of stem length and root length (cm), and plant biomass attributes (leaf, stem, and root) fresh weight (FW) (mg/plant) in two cultivar of *Brassica napus* under Ck (control), 400 μM Cr and 400 μM Cr + 1 mM GSH.

**Cultivar**	**Treatment**	**Stem length**	**Root length**	**Plant biomass**		
				**Leaf FW**	**Stem FW**	**Root FW**
ZS 758	Ck	22.14 + 2.14a	40.74 + 3.04a	0.27 + 0.02a	0.13 + 0.015a	0.152 + 0.01a
	400 μM Cr	18.66 + 2.06bc	32.52 + 2.02c	0.18 + 0.01c	0.08 + 0.011c	0.10 + 0.01c
	400 μM Cr + 1 mM GSH	20.12 + 2.02ab	36.31+ 2.01b	0.225 + 0.02b	0.106 + 0.013b	0.13 + 0.02b
Zheda 622	Ck	21.88 + 2.08a	41.03 + 2.03a	0.28 + 0.02a	0.128 + 0.01a	0.148 + 0.02a
	400 μM Cr	16.04 + 1.04c	26.47 + 2.07d	0.14 + 0.01d	0.06 + 0.012d	0.07 + 0.01d
	400 μM Cr + 1 mM GSH	18.32 + 1.02bc	29.86 + 2.06c	0.19 + 0.01c	0.088 + 0.011c	0.11 + 0.015c

*Different letters within the same column indicate statistically significant differences among the treatments following Duncan's multiple range test (P < 0.05)*.

### Cell-ultrastructure

**Figure 1:**
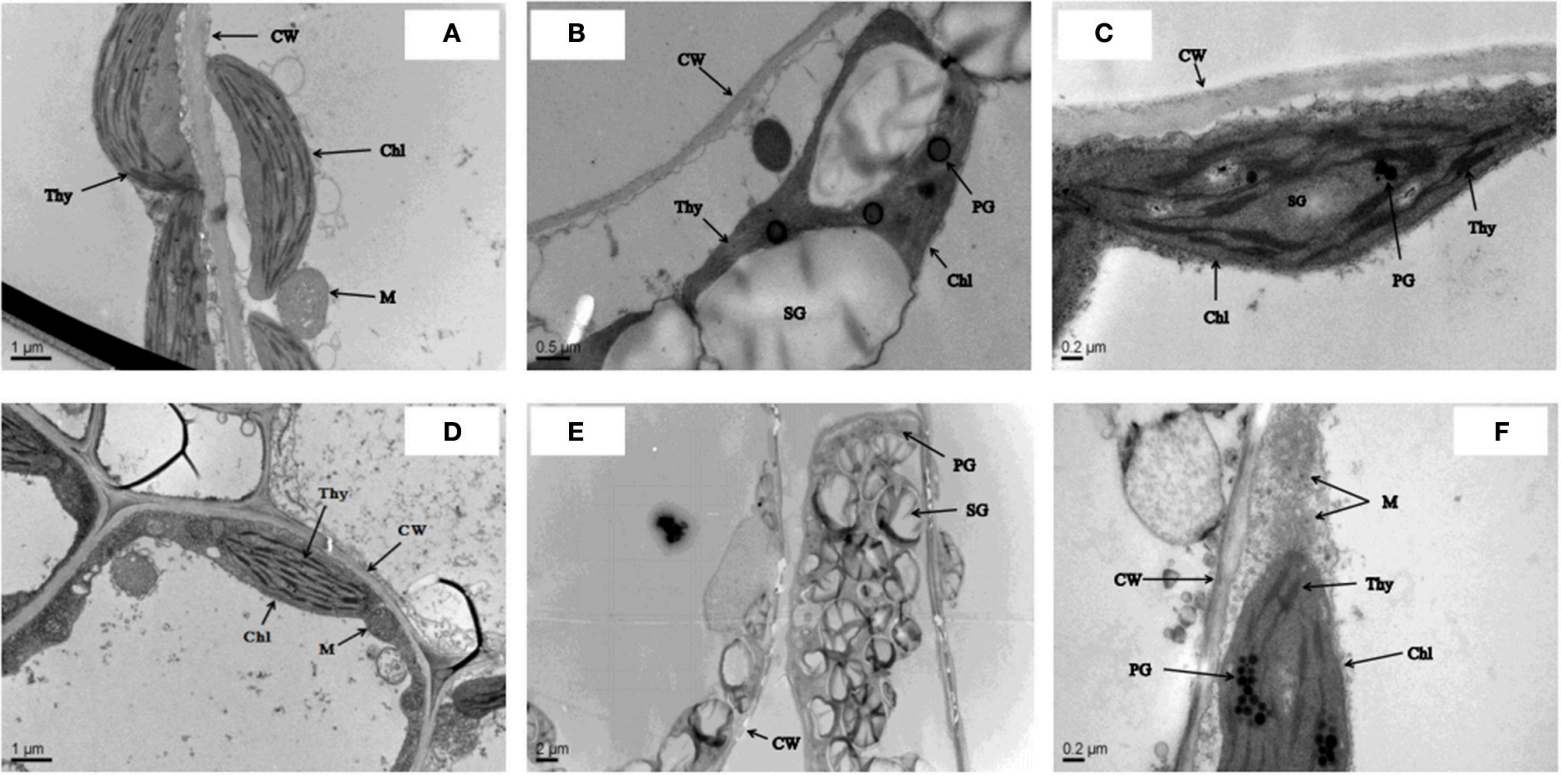
Leaf cell ultra-structural micrographs show the effect against the different treatments condition i.e., Ck (control) **(A,D)**, 400 μM Cr **(B,E)** and 400 μM Cr + 1 mM GSH **(C,F)** in two cultiavrs of *Brassica napus* i.e., ZS 758 and Zheda 622, respectively.

The ultrastructural study confirmed that cultivar ZS 758 had a clear cell wall, a typical chloroplast, mature mitochondrion, and normal thylakoid structures under control conditions (Figure 1A). Similarly, under the same growth conditions, cultivar Zheda 622 contained a smooth cell wall, a visible cristae structure in the mitochondrion, and well-shaped grana and stromal structures (Figure 1D). In a hostile environment (400 μM Cr), ZS 758 cells showed abnormal behavior in the form of large size and blackish plastoglobuli (PG), ruptured chloroplasts, and damaged thylakoid membranes (Figure 1B). Moreover, we also observed that the size of starch grains (SG) had increased, and discontinuation of the cell wall was also noticed in leaf ZS 758 mesophyll cells. Under the same hostile conditions, Zheda 622 leaf mesophyll cells were damaged badly (Figure 1E). TEM observations indicated the large SG sizes, and large numbers and sizes of PG structures. Furthermore, results highlighted that the thylakoid membranes had disappeared, and broken cell walls were also observed. However, in both cultivars, when we applied GSH to Cr-treated cells, the cells noticeably recovered from adverse organelle's changes when compared with cells that underwent only Cr-induced stress (Figures 1C,F). Moreover, results suggest that GSH improved the cell wall structure and decreased the sizes and numbers of PG and SG. Taken together, cultivar ZS 758 proved more tolerant against all of the previously described physiological and molecular parameters.

### RNA-Seq data

The raw reads of both cultivars generated by the RNA-Seq analyzer (Hi Seq 2000™) were filtered into clean reads. The raw reads that containing low-quality reads and adapters were separated (Figure S1). Figure S1 shows that the 88,630, 1,070,468, and 81,714 adapter sequences were found in ZS 758 at different levels under different conditions (control, 400 μM Cr alone, and 400 μM Cr + 1 mM GSH, respectively). Similarly, the adapter's reads in Zheda 622 were 899,129, 123,794 and 129,569 under the same treatment conditions as described above. Data regarding the low quality reads suggest that the 13114, 12814, and 16100 reads were found in ZS 758 and 18123, 18691, and 11789 reads were found in Zheda 622 under Ck, 400 μM Cr alone, and 400 μM Cr + 1 mM GSH conditions, respectively. Finally, 11874992, 10905525, and 11759780 cleans reads were found in ZS 758, and 11644490, 12509943, and 12148854 clean reads were observed in Zheda 622 under Ck, 400 μM Cr alone, and 400 μM Cr + 1 mM GSH conditions, respectively.

### Analysis of protein kinases

**Figure 2:**
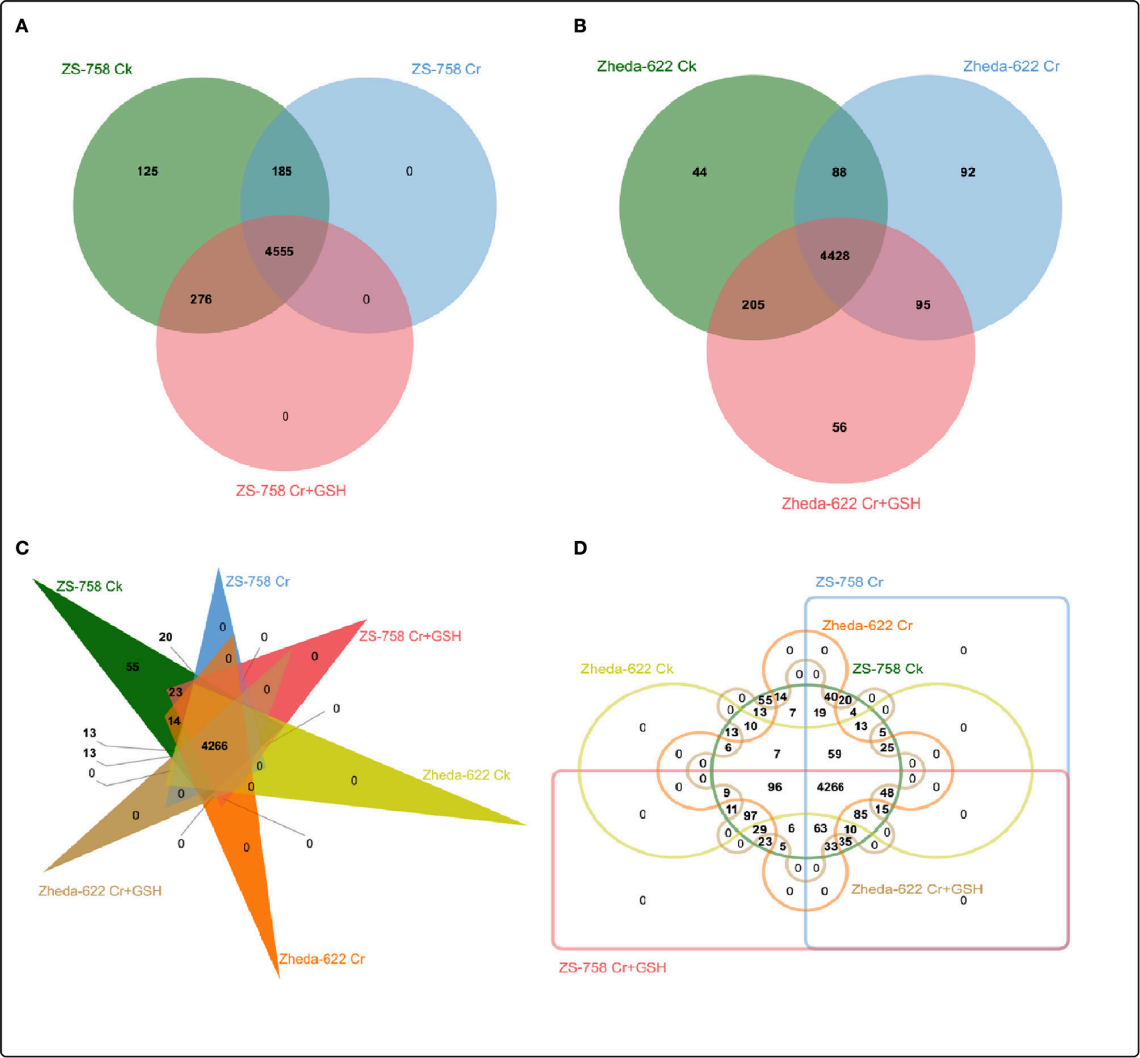
Overlapping of protein kinases among treatments between two cultivars of *Brassica napus*. **(A)** represents the ZS 758, **(B)** represents the cultivar Zheda 622, **(C,D)** represent together between cultivars and among treatments i.e., the Ck (control), Cr 400 μM, Cr 400 μM + GSH 1 mM.

After a BLAST search of the clean reads, we analyzed the part of the genome of both cultivars that responded to the protein kinases (PKs). These PKs were analyzed by Venny software (http://bioinfo.genotoul.fr/jvenn/example.html) under different treatments conditions in the two cultivars, both separately and then together (Figure 2). Results showed that the total PKs were numbered as 5141 in ZS 758 and 5008 in Zheda 622. Specifically, 125, 0, and 0 PKs were found in ZS 758 and 44, 92, and 56 in Zheda 622 under Ck, 400 μM Cr alone, and 400 μM Cr + 1 mM GSH conditions, respectively. Moreover, we also calculated the common number of PKs in the two cultivars; for instance, in ZS 758 Ck + 400 μM Cr was 185; 400 μM Cr + 400 μM Cr + 1 mM GSH was 0, Ck + 400 μM Cr + 1 mM GSH was 276, and among these all of these treatments common PKs were 4555 (Figure 2A). Similarly, in Zheda 622 the numbers of common PKs were 88, 95, 205, and 4428 under the above described conditions (Figure 2B). Furthermore, we also analyzed the two cultivars and treatments together by using the previously described Venny software (Figures 2C,D). Data showed that 55 PKs were inclusive in Ck of ZS 758 but none were found in Ck of Zheda 622 and other treatments of these two cultivars (Figure 2C). Additionally, we found 20 exclusive PKs in ZS 758, and 13 common PKs in the two cultivars under Ck conditions (Figure 2D).

#### ZS 758 as a standard

**Figure 3:**
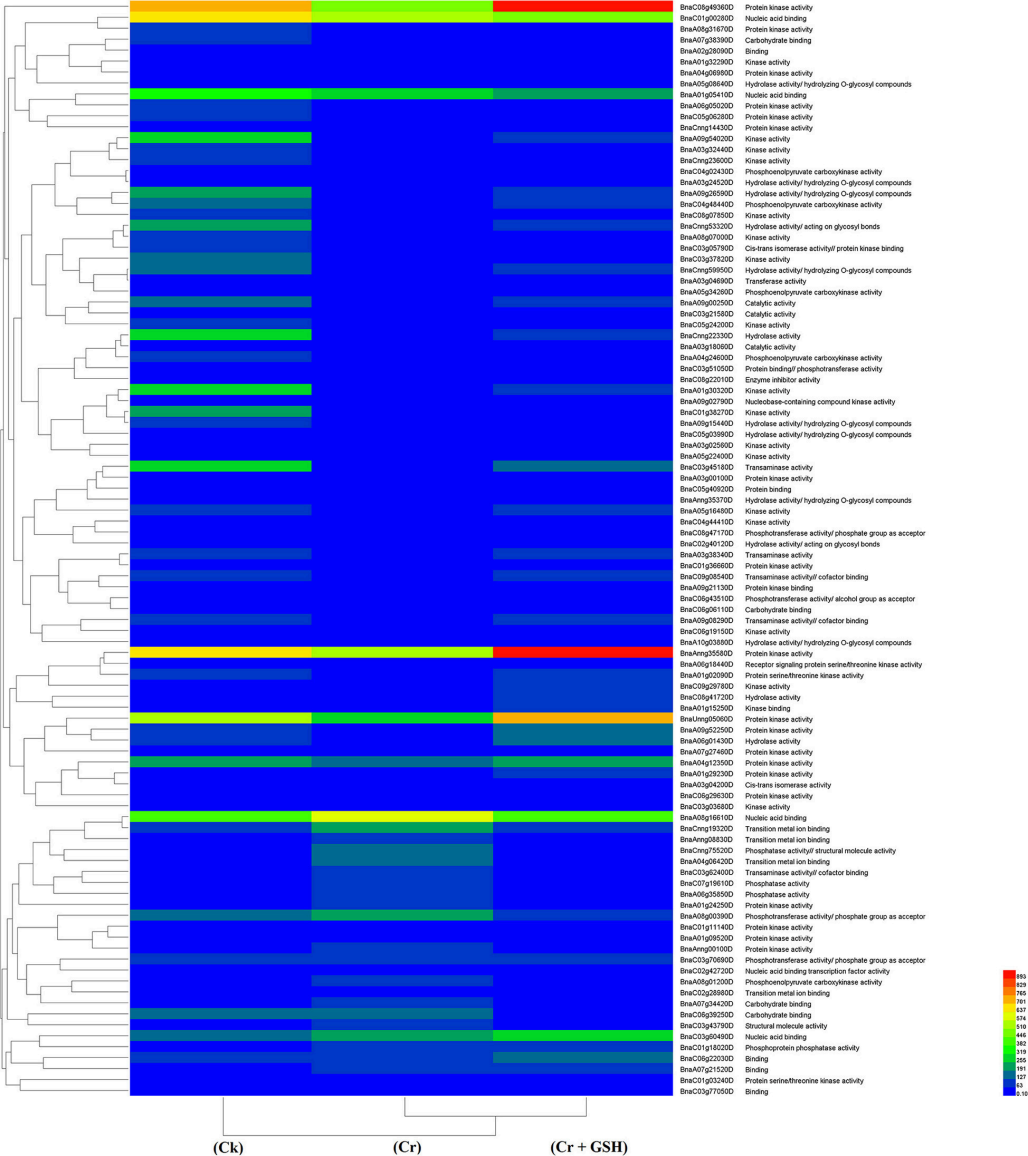
Heat map shows the protein kinases in cultivar ZS 758 of *Brassica napus* among treatments i.e., Ck (control), Cr (400 μM) and Cr + GSH (400 μM + 1 mM).

Data regarding the PKs in ZS 758 as the standard strain are presented in Table 2 (for gene coverage data, see Table S2). When we compared the cultivar ZS 758 (as the standard) with Zheda 622, several transcripts were expressed more in Zheda 622 than in Cks. These included as *BnaA09g52790D, BnaC08g49360D, BnaC01g00280D, BnaAnng35580D, BnaA09g54020D, BnaCnng22330D, BnaA01g30320D, BnaC03g45180D, BnaC01g38270D, BnaA09g26590D, BnaCnng53320D, BnaC04g48440D*, and *BnaA09g00250D* transcripts (Table 2). These transcripts were related to PK, signal transduction, and oxidoreductase activities (PKA, STA, and ORa, respectively), identical protein binding (IPb), structure-specific DNA binding (SSDNAb), nucleic acid binding (NAb), hydrolase activity (HA), hydrolysis of O-glycosyl compounds, cation and metal ion binding, trans-aminase activity, and phosphoenol pyruvate carboxylase kinase activity. Similarly, the *BnaCnng69940D, BnaA08g16610D, BnaA08g02530D*, and *BnaA04g12350D* transcripts were expressed at higher levels in Zheda 622 than in ZS 758 under the same growth conditions. The functional gene ontology (GO) of these genes was in the form of NAb, PKA, STA, ORA, IPb, and SSDNAb. Under stress conditions (400 μM Cr), both transcripts of ZS 758 with gene ID of *BnaA08g16610D* and *BnaC03g60490D* showed significant gene expression over than seen in the Cks of both cultivars (Table 2). Moreover, heat map data showed that Cr-induced cellular toxicity increased the NAb-related PKs, transition metal ion and carbohydrate binding, and phosphotransferase, phosphatase, and structural molecular activities when compared with Cks (Figure 3). When we added GSH to the Cr solution, the *BnaAnng35580D, BnaUnng05060D* (PKA, ST, ORA, IPb, and SSDNAb), and *BnaCnng69940D* transcripts were expressed more when compared with Cks of both cultivars. Moreover, transcripts named with gene IDs of *BnaC08g49360D*, and *BnaA04g12350D* (PKA, STA, ORA, IPb, and SSDNAb) showed higher expression in Zheda 622. However, we did not find any transcripts that showed increased expression in Zheda 622 under Cr-induced stress conditions (Table 2). Additionally, heat map results highlighted that NAb and transaminase, HA, and PKA were increased after GSH addition over that seen under conditions of Cr alone (Figure 2).

**Table 2:** Transcripts related to protein kinases (RPKM) under the different treatment conditions i.e., Ck (control), 400 μM Cr and 400 μM Cr + 1 mM GSH in two cultivars of *Brassica napus*, while Ck (ZS 758) used as a standard.

**Gene ID**	**ZS 758**			**Zheda 622**			**Gene description (GO Function)**
	**Ck**	**Cr**	**Cr + GSH**	**Ck**	**Cr**	**Cr + GSH**	
*BnaA09g52790D*	1,059.25	61.37	439.47	183.89	14.28	176.84	–
*BnaC08g49360D*	757.12	491.87	956.92	673.17	524.31	1,251.69	Protein kinase activity (PKA); signal transducer activity (STA); Oxido-reductase activity (ORA), acting on the aldehyde or oxo group of donors, NAD or NADP as acceptor; identical protein binding (IPb); structure-specific DNA binding (SSDNAb).
*BnaC01g00280D*	685.35	546.76	501.25	271.99	281.99	271.01	Nucleic acid binding (NAb); PKA; STA; ORA; IPb.
*BnaAnng35580D*	666.41	519.17	946.34	168.04	112.80	345.04	PKA; STA; ORA; IPb; SSDNAb.
*BnaUnng05060D*	519.10	259.66	701.78	207.44	114.59	386.09	PKA; STA; ORA; IPb; SSDNAb.
*BnaCnng69940D*	414.34	105.09	1,083.69	544.78	106.66	1,040.18	–
*BnaA08g16610D*	383.64	577.39	390.69	534.05	463.94	617.15	NAb; PKA; STA; ORA; IPb.
*BnaA01g05410D*	357.54	274.40	195.37	347.08	256.82	248.19	NAb; PKA; STA; ORA; IPb.
*BnaA09g54020D*	309.23	0.69	117.30	190.49	0.84	64.85	Kinase activity (KA).
*BnaCnng22330D*	295.81	14.01	91.30	39.39	3.59	42.04	Hydrolase activity (HA), hydrolyzing O-glycosyl compounds; cation binding.
*BnaA01g30320D*	278.27	3.46	63.60	125.37	4.03	44.02	KA.
*BnaC03g45180D*	269.99	0.85	183.14	236.93	0.68	127.84	Binding; trans-aminase activity.
*BnaC01g38270D*	216.84	3.58	51.65	109.96	4.07	37.40	KA.
*BnaA08g02530D*	216.56	14.96	100.71	317.46	24.69	117.25	–
*BnaA04g12350D*	212.78	130.34	240.95	302.75	176.17	542.05	PKA; STA; ORA; IPb; SSDNAb.
*BnaA09g26590D*	209.22	25.45	86.62	58.81	11.04	34.06	HA, hydrolyzing O-glycosyl compounds; cation binding (Cb).
*BnaCnng53320D*	199.12	20.72	93.03	65.55	8.08	49.60	HA, acting on glycosyl bonds.
*BnaC03g60490D*	164.01	204.42	288.83	244.41	176.04	318.45	NAb; PKA; STA; ORA; IPb.
*BnaC04g48440D*	159.56	19.72	65.72	110.61	7.26	48.69	Metal ion binding; phosphoenol pyruvate carboxylase kinase activity.
*BnaA09g00250D*	159.46	1.39	77.73	73.77	1.38	59.94	Binding; catalytic activity.

*Green-white-red color scale shows the values from highest level to lowest*.

#### Zheda 622 as a standard

**Figure 4:**
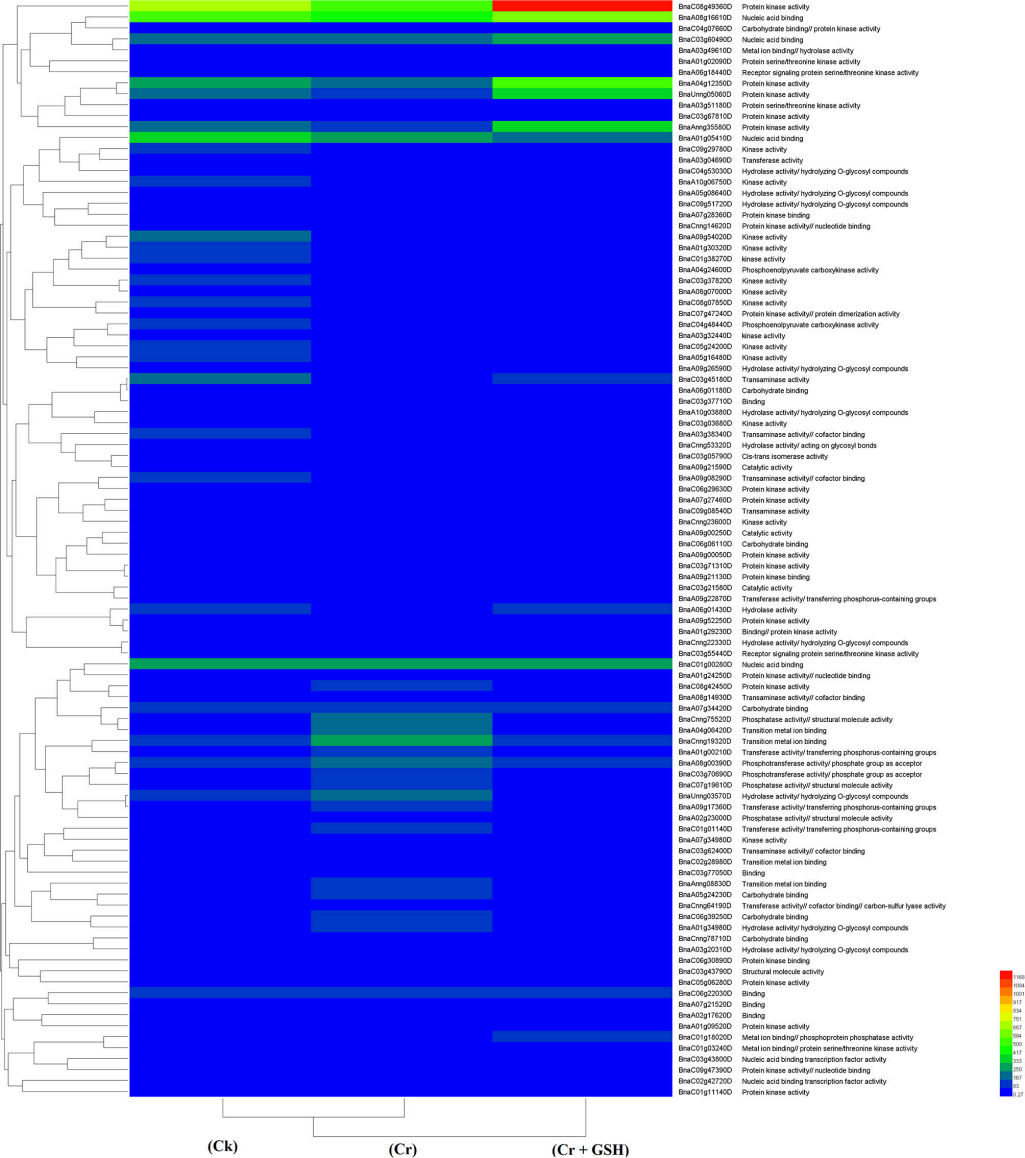
Heat map shows the protein kinases in cultivars Zheda 622 of *Brassica napus* among treatments i.e., Ck (control), Cr (400 μM) and Cr + GSH (400 μM + 1 mM).

Data regarding PKs in both cultivars (using Zheda 622 as a standard) under the different treatments conditions are shown in Table 3 (for gene coverage data, see Table S3). The *BnaC08g49360D, BnaA01g05410D, BnaC01g00280D, BnaC03g45180D, BnaUnng05060D, BnaA09g54020D*, and *BnaAnng35580D* transcripts were expressed more in ZS 758 under Ck conditions. These transcripts were related to PKA, STA, NAb, IPb, SSDNAb, and transaminase, kinase, and ORA activities. Similarly, in Zheda 622, the *BnaA08g16610D, BnaA10g05680D, BnaA04g12350D, BnaC09g29780D*, and *BnaCnng19320D* genes were expressed at higher levels when compared with the ZS 758 Ck levels. Under toxic conditions (400 μM Cr), the *BnaA08g16610D* (NAb, PKA) and *BnaCnng19320D* transcripts were expressed at a higher levels in ZS 758 than in Ck cells in both cultivars. Interestingly, *BnaA08g00390D* (containing cation binding and phospho-transferase activity) was expressed at same level in both cultivars under Cr-induced stress conditions and was higher than Ck in both cultivars (Table 3). Furthermore, heat map data determined that HA and transferase activities were increased under toxic Cr conditions when compared with Ck (Figure 4). After addition of GSH into the solution, the *BnaAnng35580D* and *BnaC09g29780D* (both described above) transcripts were expressed more in ZS 758 than under Ck and Cr-induced stress conditions in both cultivars. Similarly, the *BnaC08g49360D, BnaA08g16610D* (both described above) and *BnaA04g12350D* (PKA; SSDNAb) transcripts were significantly expressed more in Zheda 622 in the presence of GSH when compared with Ck and Cr-induced stress conditions in both cultivars. Moreover, heat map data showed that PKA, metal ion binding, phosphor-protein phosphatase activity, and NAb-related transcripts were increased in Zheda 622 exposed to the combined treatment (Cr + GSH) (Figure 4). In general, when we examined either ZS 758 or Zheda 622 as a standard, cultivar ZS 758 performed better.

**Table 3:** Transcripts related to protein kinases (RPKM) under the different treatment conditions i.e., Ck (control), 400 μM Cr and 400 μM Cr + 1 mM GSH in two cultivars of *Brassica napus*, while Ck (Zheda 622) used as a standard.

**Gene ID**	**ZS 758**			**Zheda 622**			**Gene description (GO Function)**
	**CK**	**Cr**	**Cr + GSH**	**CK**	**Cr**	**Cr + GSH**	
*BnaC08g49360D*	757.12	491.87	956.92	673.17	524.31	1251.69	Protein kinase activity (PKA); signal transducer activity (STA).
*BnaA08g16610D*	383.64	577.39	390.69	534.05	463.94	617.15	Nucleic acid binding (NAb); PKA.
*BnaA10g05680D*	0.00	0.29	42.02	414.14	9.53	129.89	Hydrolase activity (HA), hydrolyzing O-glycosyl compounds.
*BnaA01g05410D*	357.54	274.40	195.37	347.08	256.82	248.19	NAb; PKA.
*BnaA04g12350D*	212.78	130.34	240.95	302.75	176.17	542.05	PKA; STA; structure-specific DNA binding (SSDNAb).
*BnaC01g00280D*	685.35	546.76	501.25	271.99	281.99	271.01	NAb; PKA; identical protein binding (IPb).
*BnaC03g60490D*	164.01	204.42	288.83	244.41	176.04	318.45	NAb; PKA; IPb.
*BnaC03g45180D*	269.99	0.85	183.14	236.93	0.68	127.84	Binding; transaminase activity.
*BnaUnng05060D*	519.10	259.66	701.78	207.44	114.59	386.09	PKA; STA; SSDNAb.
*BnaA09g54020D*	309.23	0.69	117.30	190.49	0.84	64.85	Kinase activity.
*BnaAnng35580D*	666.41	519.17	946.34	168.04	112.80	345.04	PKA; STA; oxido-reductase activity.
*BnaC09g29780D*	48.40	21.43	88.40	145.97	52.72	49.83	Binding; kinase activity.
*BnaCnng19320D*	75.23	217.40	80.24	134.27	272.39	89.01	Transition metal ion binding.
*BnaA01g30320D*	278.27	3.46	63.60	125.37	4.03	44.02	Kinase activity.
*BnaA08g00390D*	129.17	197.75	103.62	116.14	202.02	100.49	Cation binding; phospho-transferase activity.
*BnaA06g01430D*	87.03	2.37	141.74	116.04	2.16	153.25	Hydrolase activity.
*BnaC04g48440D*	159.56	19.72	65.72	110.61	7.26	48.69	Metal ion binding; phosphoenolpyruvate carboxykinase activity.
*BnaC01g38270D*	216.84	3.58	51.65	109.96	4.07	37.40	Kinase activity.
*BnaA05g16480D*	110.94	0.10	69.70	102.29	0.27	46.68	Kinase activity.
*BnaC03g37820D*	132.33	7.26	60.69	101.89	10.18	36.35	Kinase activity.

*Green-yellow color scale shows the values from highest level to lowest*.

### Exploration of metal stress related protein kinases

In order to be more pragmatic about the role of PKs with regard to metal stress, we concurrently analyzed metal stress in two cultivars under the three previously described treatments (Table 4). Results showed that main classes of metal stress-related PKs included both metal ion and transition metal ion binding, transaminase activity, ion channels, small conjugating protein ligase activity, and calmodulin-and cyclin-dependent PKs. Data showed that metal ion binding further classified to hydrolase activity, inositol trisphosphate kinase activity; inositol tetra-kisphosphate kinase activity, phosphatidylinositol phosphate kinase activity, protein kinase activity, protein kinase C activity and protein serine/threonine kinase activity. Similarly, the transition metal ion binding were subdivided to transaminase activity, PK binding, adenosine deaminase activity, kinase activity and small GTPase regulator activity, 4-aminobutyrate transaminase activity and hydrolase activity, acting on carbon-nitrogen (but not peptide) bonds, in cyclic amides.

**Table 4:** Represented the genes regarding the protein kinases (RPKM) that related to metal stress in two *Brassica napus* cultivars under the different treatment conditions i.e., Ck (control), 400 μM Cr and 400 μM Cr + 1 mM GSH.

**Gene ID**	**ZS 758**			**Zheda 622**			**GO Function**
	**Ck**	**Cr**	**Cr + GSH**	**Ck**	**Cr**	**Cr + GSH**	
**METAL ION BINDING**							
*BnaAnng03390D*	2.78	3.82	4.50	4.06	3.77	4.93	Metal ion binding
*BnaA03g08660D*	1.85	2.01	3.37	3.78	2.40	5.37	Metal ion binding
*BnaA03g49610D*	30.01	17.54	40.06	34.87	24.95	45.90	Metal ion binding; hydrolase activity
*BnaC09g41080D*	3.37	5.57	4.06	6.66	5.65	4.87	Metal ion binding; inositol trisphosphate kinase activity; inositol tetrakisphosphate kinase activity
*BnaA03g06170D*	1.25	2.51	2.86	4.11	3.11	2.45	Metal ion binding; inositol trisphosphate kinase activity; inositol tetrakisphosphate kinase activity
*BnaC01g04950D*	3.74	4.27	4.30	3.76	2.69	5.73	Metal ion binding; phosphatidylinositol phosphate kinase activity
*BnaC04g48440D*	159.56	19.72	65.72	110.61	7.26	48.69	Metal ion binding; phosphoenolpyruvate carboxykinase activity
*BnaA04g24600D*	92.71	4.80	29.16	45.82	1.65	17.45	Metal ion binding; phosphoenolpyruvate carboxykinase activity
*BnaC04g02430D*	46.39	5.03	19.68	23.17	2.51	16.52	Metal ion binding; phosphoenolpyruvate carboxykinase activity
*BnaC06g09980D*	28.09	58.93	12.90	6.50	26.30	13.11	Metal ion binding; phosphoenolpyruvate carboxykinase activity
*BnaA06g19580D*	5.17	7.35	7.17	5.93	5.99	8.21	Metal ion binding; phospholipid binding
*BnaA01g00260D*	8.80	1.06	5.00	7.46	2.83	5.63	Metal ion binding; protein kinase activity
*BnaC08g36980D*	4.24	2.81	2.90	5.37	3.51	4.35	Metal ion binding; protein kinase activity
*BnaA07g35090D*	3.22	1.56	9.76	3.41	2.30	5.29	Metal ion binding; protein kinase activity
*BnaA10g25410D*	3.04	9.25	3.81	3.21	7.56	3.05	Metal ion binding; protein kinase activity
*BnaC01g33560D*	1.33	3.15	2.11	2.21	5.64	3.64	Metal ion binding; protein kinase activity
*BnaA07g17350D*	5.46	7.89	11.16	6.21	6.22	10.65	Metal ion binding; protein kinase C activity
*BnaA09g36820D*	2.74	3.98	3.30	1.99	3.47	2.84	Metal ion binding; protein kinase C activity
*BnaCnng24360D*	1.50	0.91	2.59	0.00	0.00	0.00	Metal ion binding; protein kinase C activity
*BnaA03g41500D*	4.99	6.56	5.47	4.08	6.01	4.85	Metal ion binding; protein serine/threonine kinase activity
*BnaA06g26290D*	1.97	3.52	2.50	2.21	2.57	2.44	Metal ion binding; protein serine/threonine kinase activity
*BnaA03g53230D*	1.57	2.48	3.37	3.33	3.88	5.00	Metal ion binding; protein serine/threonine kinase activity
*BnaC09g04790D*	8.67	7.67	19.44	15.42	8.52	20.39	Metal ion binding; protein serine/threonine kinase activity
*BnaA09g05220D*	8.26	3.61	17.57	8.88	2.86	14.26	Metal ion binding; protein serine/threonine kinase activity
**TRANSITION METAL ION BINDING**							
*BnaC02g28980D*	32.81	41.04	23.15	34.13	51.94	34.32	Transition metal ion binding
*BnaA08g11470D*	13.40	9.30	18.39	11.85	7.60	10.07	Transition metal ion binding; transaminase activity
*BnaA07g06940D*	10.51	2.49	8.42	7.80	2.94	7.64	Transition metal ion binding; protein kinase binding
*BnaC02g18410D*	10.42	8.03	14.25	8.41	8.36	12.95	Transition metal ion binding; adenosine deaminase activity
*BnaCnng72730D*	8.56	2.59	10.45	11.34	1.35	13.22	Transition metal ion binding; transaminase activity
*BnaA02g01550D*	7.04	0.55	5.66	4.26	0.39	8.46	Transition metal ion binding; kinase activity; small GTPase regulator activity
*BnaA01g24820D*	3.12	4.46	9.31	3.72	5.38	7.69	Transition metal ion binding; 4-aminobutyrate transaminase activity
*BnaA01g30570D*	2.98	8.07	14.52	7.52	6.99	10.77	Transition metal ion binding; endopeptidase inhibitor activity
*BnaA05g17430D*	2.66	1.51	4.84	3.27	1.67	5.00	Transition metal ion binding; 4-aminobutyrate transaminase activity
*BnaA10g15590D*	1.93	6.65	2.48	4.28	7.31	2.88	Transition metal ion binding; transaminase activity; cofactor binding
*BnaA09g20120D*	1.52	2.29	6.80	5.14	1.81	8.47	Transition metal ion binding; hydrolase activity, acting on carbon-nitrogen (but not peptide) bonds, in cyclic amides
**TRANSAMINASE ACTIVITY**							
*BnaC03g45180D*	269.99	0.85	183.14	236.93	0.68	127.84	Transaminase activity
*BnaA03g38340D*	107.28	0.51	84.81	88.03	0.30	58.15	Transaminase activity; cofactor binding
*BnaA03g03400D*	4.99	6.23	9.91	3.62	4.99	5.55	Transaminase activity; cofactor binding
*BnaA09g10660D*	4.74	3.72	6.62	7.11	3.53	6.70	Transaminase activity
**ION CHANNEL**							
*BnaA03g03770D*	2.10	3.75	1.88	1.50	3.66	1.44	Ion channel activity
*BnaC03g05320D*	2.59	1.41	5.14	4.04	2.07	4.90	Ion channel activity
*BnaC09g44590D*	7.93	3.68	7.21	10.05	4.46	10.80	Ion channel activity
**SMALL CONJUGATING PROTEIN LIGASE ACTIVITY**							
*BnaA05g27260D*	0.98	0.87	2.22	3.60	1.53	2.91	Small conjugating protein ligase activity
*BnaC05g00400D*	1.36	2.34	5.19	1.75	1.85	1.77	Small conjugating protein ligase activity
*BnaCnng13500D*	2.53	3.05	5.94	3.71	2.70	6.08	Small conjugating protein ligase activity
*BnaA06g18030D*	8.90	12.95	14.00	8.14	9.14	8.22	Small conjugating protein ligase activity; receptor serine/threonine kinase binding;
**CALMODULIN- AND CYCLIN-DEPENDENT PROTEIN KINASE ACTIVITY**							
*BnaA02g20780D*	10.02	7.95	8.64	9.18	8.80	7.65	Calmodulin-dependent protein kinase activity
*BnaC02g27270D*	2.83	5.91	6.64	5.59	5.92	7.98	Calmodulin-dependent protein kinase activity
*BnaC05g15880D*	1.82	4.40	3.04	1.93	3.34	2.99	Calmodulin-dependent protein kinase activity
*BnaCnng04940D*	1.51	2.13	3.38	3.73	1.75	4.22	Calmodulin-dependent protein kinase activity
*BnaCnng63340D*	0.57	2.46	1.52	1.32	0.85	1.78	Calmodulin-dependent protein kinase activity
*BnaA02g03250D*	0.23	0.87	0.47	0.28	0.70	0.32	Calmodulin-dependent protein kinase activity
*BnaC03g73980D*	2.46	5.74	5.08	1.91	6.67	4.84	Cyclin-dependent protein kinase regulator activity; protein binding
*BnaC06g02810D*	1.00	0.52	1.93	1.22	0.77	1.27	Cyclin-dependent protein kinase regulator activity; protein binding

### Analysis of molecular transporters

**Figure 5:**
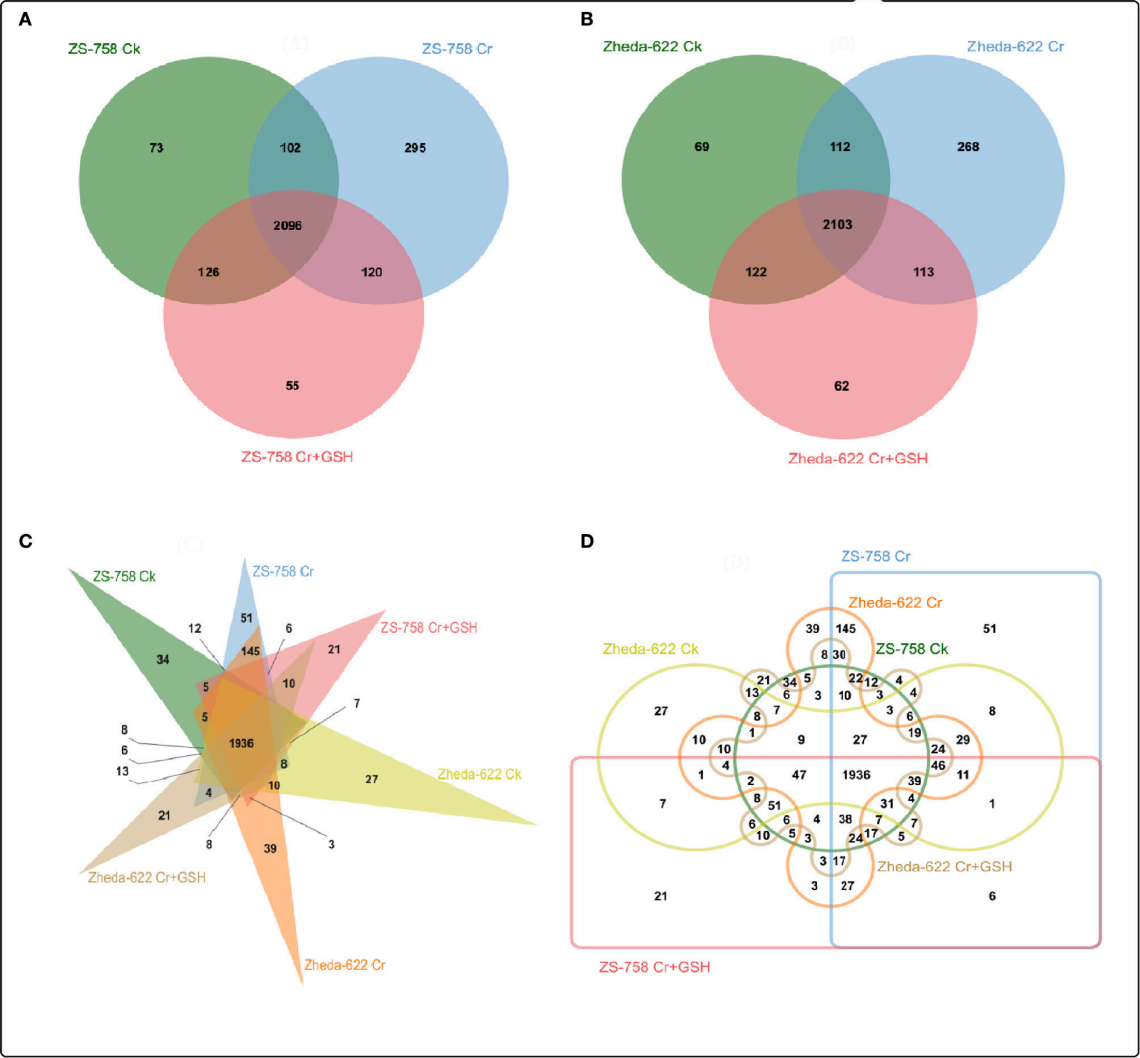
Show the overlapping of molecular transporters among treatments between two cultivars of *Brassica napus*. **(A)** represents the ZS 758, **(B)** represents the cultivar Zheda 622, **(C,D)** represent together between cultivars and among treatments i.e., the Ck (control), Cr 400 μM, Cr 400 μM +GSH 1 mM.

We also analyzed the molecular transporters (MTs) in both cultivars that numbered 2867 in ZS758 and 2849 in Zheda 622 (Figure 5). Specifically, we observed 73, 295, and 55 MTs in ZS 758 and 69, 268, and 62 in Zheda 622 under Ck, 400 μM Cr alone, and 400 μM Cr + 1 mM GSH conditions, respectively. Moreover, we also calculated the number of overlapping transporters in both cultivars and under the three different treatment; for instance, in ZS 758, Ck + 400 μM Cr was 102; 400 μM Cr + (400 μM Cr + 1 mM GSH) was 120, Ck + (400 μM Cr + 1 mM GSH) was 126, and 2096 were common among all treatments (Figure 5A). Similarly, overlapping molecular transporters in Zheda 622 were 112, 113, 122, and 2,103 under the previously described conditions (Figure 5B). We also calculated the MTs in the two cultivars and under the three treatments (Figures 5C,D). Results showed that 34, 51, and 21 MTs were included exclusively in ZS 758 and 27, 39, and 21 were in Zheda 622 under the Ck, Cr, and Cr + GSH conditions (Figure 5C). Moreover, Figure 5D indicated that 8, 145, and 3 MTs were common in both cultivars under the three different conditions.

#### ZS 758 as a standard

**Figure 6:**
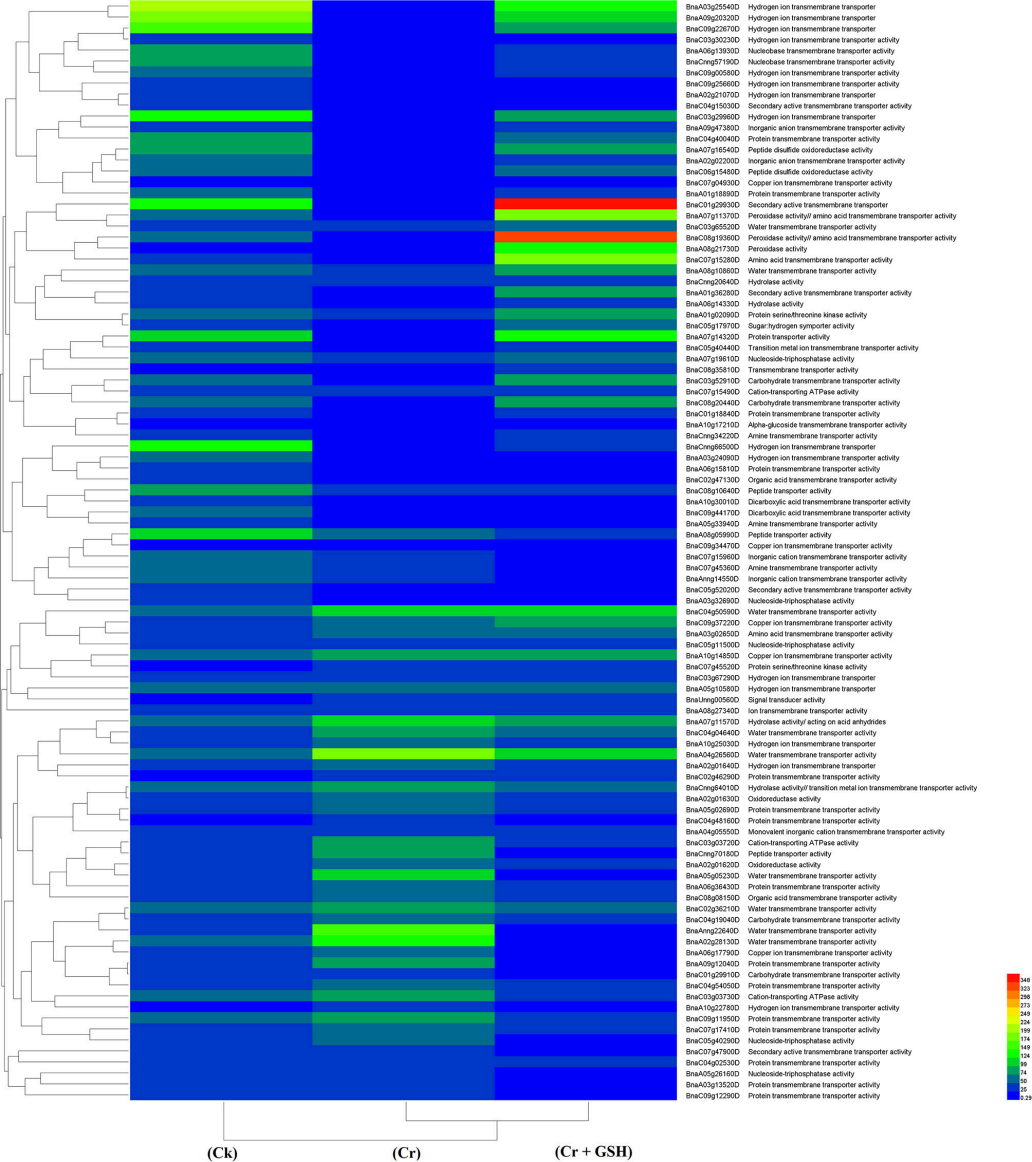
Heat map shows the molecular transporters in cultivar ZS 758 of *Brassica napus* among treatments i.e., Ck (control), Cr (400 μM) and Cr + GSH (400 μM + 1 mM).

Similar to protein kinases, we analyzed the molecular transporters in cultivar ZS 758 (Table 5, for gene coverage data, see Table S4). Results showed that under Ck conditions, the *BnaC07g15280D, BnaC03g29960D, BnaA08g21730D, BnaCnng66500D*, and *BnaA07g14320D* transcripts were expressed at higher levels in ZS 758 when compared with Zheda 622. These genes were related to peroxidase activity; amino acid trans-membrane transporter activity; iron ion binding, hydrogen ion transmembrane transporter activity; cation-transporting ATPase activity, and protein transporter activity. Under stress conditions (400 μM Cr), the *BnaA04g26560D* and *BnaA02g28130D* transcripts were expressed and corresponded to water transmembrane transporter activity, which was found in the ZS 758 cultivar (Table 5). The *BnaA04g26560D* transcript was also expressed in Zheda 622 under Cr-induced stress conditions. However, we did not find higher levels of genes that would alleviate Cr toxicity in Zheda 622. Heat map data showed that MTs related to hydrolase trans-membrane transporter and protein transporter activities were increased under Cr-induced stress conditions over that seen in Ck (Figure 6). Further, when GSH was added to the medium, the *BnaA07g14320D* transcript (described above) was increased in ZS 758 when compared with both Cr-induced stress conditions and Ck (Table 5). Moreover, at this level *BnaC08g19360D* and *BnaA07g11370D* transcripts were also higher in Zheda 622 when compared with both Cr-induced stress and Ck conditions. These two genes were related to peroxidase and amino acid trans-membrane transporter activities and Fe binding. Moreover, when compared with Cr alone, heat map results showed that GSH in combination with Cr also increased the MT expressions, which corresponded to H+ ion trans-membrane transporter activity (Figure 6).

**Table 5:** Analysis of transcripts coding the molecular transporters (RPKM) under the different treatment conditions i.e., Ck (control), 400 μM Cr and 400 μM Cr + 1 mM GSH in two cultivars of *Brassica napus*, while Ck (ZS 758) used as a standard.

**Gene ID**	**ZS 758**			**Zheda 622**			**Gene description (GO Function)**
	**Ck**	**Cr**	**Cr + GSH**	**Ck**	**Cr**	**Cr + GSH**	
*BnaC07g15280D*	315.81	29.43	239.51	42.97	13.27	188.46	Peroxidase activity; amino acid trans-membrane transporter activity; iron ion binding.
*BnaC03g29960D*	251.98	1.83	142.12	142.98	2.27	95.14	Hydrogen ion trans-membrane transporter activity; cation-transporting ATPase activity.
*BnaA08g21730D*	247.75	31.17	112.01	24.03	6.58	141.00	Peroxidase activity; amino acid trans-membrane transporter activity; iron ion binding.
*BnaCnng66500D*	236.80	1.11	59.46	127.95	2.94	40.16	Hydrogen ion trans-membrane transporter activity.
*BnaC08g19360D*	217.22	17.75	168.06	58.81	14.53	323.48	Peroxidase activity; amino acid trans-membrane transporter activity; iron ion binding.
*BnaA03g25540D*	203.96	0.73	115.01	220.44	2.81	137.98	Hydrogen ion trans-membrane transporter activity; cation-transporting ATPase activity.
*BnaA07g14320D*	194.99	9.90	232.21	117.99	13.01	140.71	Protein transporter activity.
*BnaC09g22670D*	184.36	2.67	100.21	166.56	2.36	86.79	Hydrogen ion trans-membrane transporter activity; cation-transporting ATPase activity.
*BnaA09g20320D*	173.87	2.41	124.33	183.32	2.58	113.51	Hydrogen ion trans-membrane transporter activity; cation-transporting ATPase activity.
*BnaA04g26560D*	118.38	181.27	92.92	62.72	181.76	102.18	Water trans-membrane transporter activity.
*BnaA02g28130D*	116.95	153.35	33.90	54.40	135.11	23.19	Water trans-membrane transporter activity.
*BnaC04g40040D*	113.59	11.88	101.81	96.76	11.85	72.73	Binding; protein trans-membrane transporter activity.
*BnaA08g10860D*	110.30	59.24	87.63	66.84	49.77	92.90	Water trans-membrane transporter activity.
*BnaC02g36210D*	109.68	89.11	50.62	65.08	85.33	60.17	Water trans-membrane transporter activity.
*BnaC09g25660D*	100.21	2.52	23.90	40.19	2.64	18.50	Hydrogen ion trans-membrane transporter activity.
*BnaA07g11370D*	98.66	20.51	157.57	68.50	19.53	188.85	Peroxidase activity; amino acid trans-membrane transporter activity; iron ion binding.
*BnaA07g16540D*	98.44	21.59	85.60	90.87	23.69	77.24	Peptide disulfide oxido-reductase activity.
*BnaA03g24090D*	96.11	0.27	26.44	52.80	0.97	19.19	Hydrogen ion trans-membrane transporter activity.
*BnaA02g21070D*	95.31	5.08	35.40	36.85	6.60	19.93	Hydrogen ion trans-membrane transporter activity.
*BnaC07g45360D*	86.24	31.76	39.48	50.51	25.06	15.87	Amine trans-membrane transporter activity; peroxidase activity; iron ion binding.

*Green-white color scale shows the values from highest to lowest*.

#### Zheda 622 as a standard

**Figure 7:**
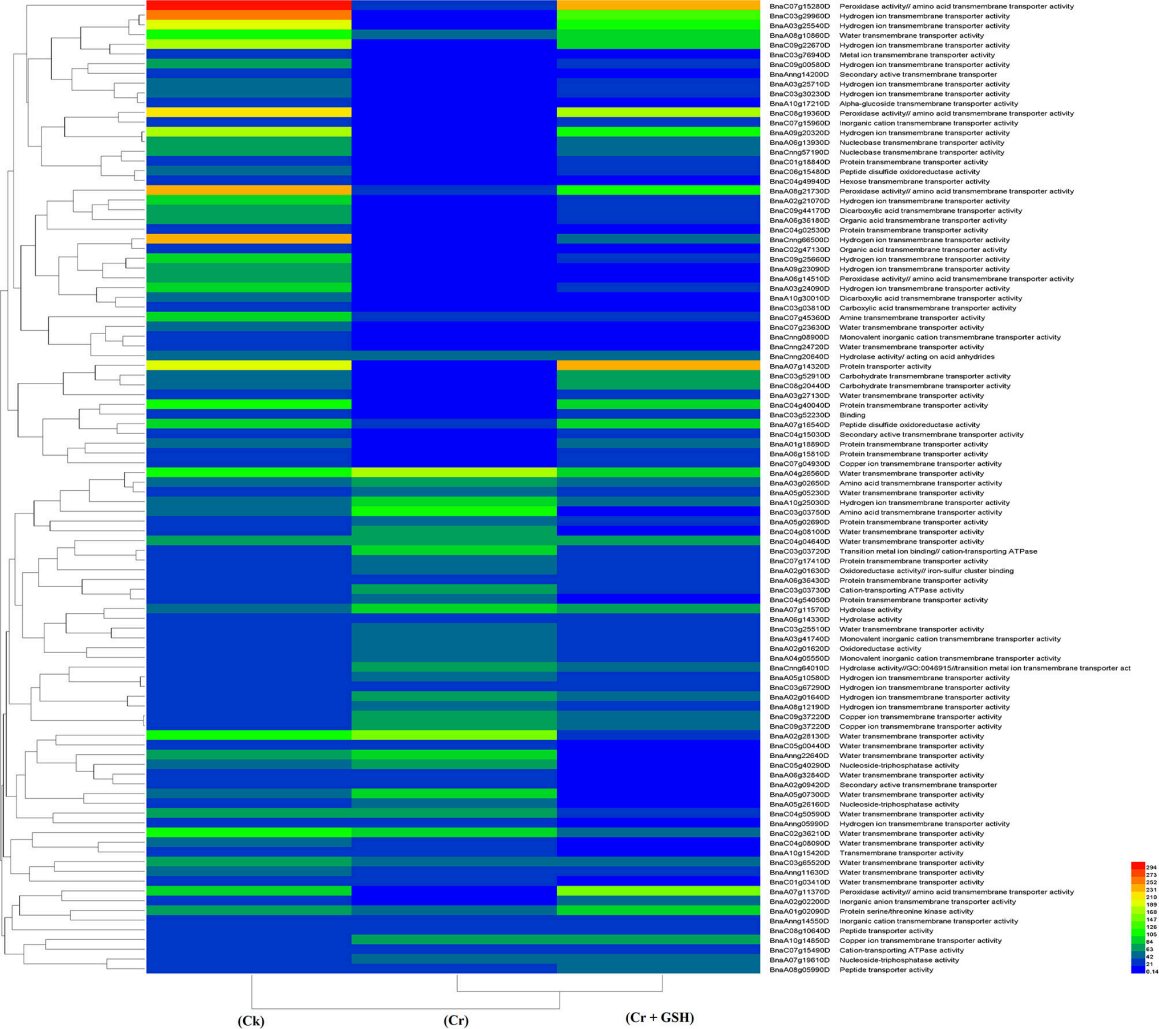
Heat map shows the molecular transporters in cultivar Zheda 622 of *Brassica napus* among treatments i.e., Ck (control), Cr (400 μM) and Cr + GSH (400 μM + 1 mM).

When the cultivar Zheda 622 was taken as the standard, the MTs and *BnaC09g22670D, BnaC03g29960D*, and *BnaCnng66500D* transcripts were increased in ZS 758 in Cks (Table 6, for gene coverage data, see Table S5). These genes were related to hydrogen ion trans-membrane transporter and cation-transporting ATPase activities. Similarly, in Zheda 622 the *BnaA03g25540D* and *BnaA09g20320D* transcripts were expressed at higher levels when compared with Ck in ZS 758. The GO functions of these transcripts were was similar to that seen in ZS758. At 400 μM Cr, *BnaA02g01980D* (unknown) gene expression was increased in ZS 758 when compared with Cks and combined GSH and Cr treatment in both cultivars. In a similar manner, the *BnaC04g50590D* transcript, which corresponded to water trans-membrane transporter activity, was increased in Zheda 622. Under Cr-induced stress conditions, heat map data showed a Zheda 622 exclusive increase in transcript expressions that were related to Cu+ ion trans-membrane transporter and AA transmembrane transporter activities (Figure 7). When GSH was added to the media, *BnaC01g29930D, BnaA07g11370D* and *BnaA07g14320D* genes, which were related to iron binding and secondary active transmembrane transporter, organic acid transmembrane transporter, peroxidase, amino acid transmembrane transporter, and protein transporter activities, were increased in both cultivars when compared with the cultivars under Cr or Ck conditions. Moreover, heat map results showed that MTs related to protein transporter, protein serine/threonine kinase, and amine trans-membrane transporter activities were increased by GSH and Cr in combination than by Cr alone (Figure 7).

**Table 6:** Transcripts related to molecular transporters (RPKM) under the different treatment conditions i.e., Ck (control), 400 μM Cr and 400 μM Cr + 1 mM GSH in two cultivars of *Brassica napus*, while Ck (Zheda 622) used as a standard.

**Gene ID**	**ZS 758**			**Zheda 622**			**Gene description (GO Function)**
	**Ck**	**Cr**	**Cr + GSH**	**Ck**	**Cr**	**Cr + GSH**	
*BnaA03g25540D*	203.96	0.73	115.01	220.44	2.81	137.98	Hydrogen ion trans-membrane transporter activity; cation-transporting ATPase activity.
*BnaA09g20320D*	173.87	2.41	124.33	183.32	2.58	113.51	Hydrogen ion trans-membrane transporter activity; cation-transporting ATPase activity.
*BnaC09g22670D*	184.36	2.67	100.21	166.56	2.36	86.79	Hydrogen ion trans-membrane transporter activity; cation-transporting ATPase activity.
*BnaC03g29960D*	251.98	1.83	142.12	142.98	2.27	95.14	Hydrogen ion trans-membrane transporter activity; cation-transporting ATPase activity.
*BnaC01g29930D*	8.41	5.03	311.36	134.66	6.82	373.40	Secondary active trans-membrane transporter activity; organic acid trans-membrane transporter activity.
*BnaCnng66500D*	236.80	1.11	59.46	127.95	2.94	40.16	Hydrogen ion trans-membrane transporter activity.
*BnaA08g05990D*	28.89	38.57	42.99	120.88	51.81	32.43	Peptide transporter activity.
*BnaA07g14320D*	194.99	9.90	232.21	117.99	13.01	140.71	Protein transporter activity.
*BnaC04g40040D*	113.59	11.88	101.81	96.76	11.85	72.73	Binding; Protein trans-membrane transporter activity.
*BnaA06g13930D*	69.17	3.04	50.02	94.63	3.36	48.22	Nucleo-base trans-membrane transporter activity.
*BnaA07g16540D*	98.44	21.59	85.60	90.87	23.69	77.24	Peptide disulfide oxido-reductase activity; Secondary active trans-membrane transporter activity.
*BnaA02g01980D*	59.67	142.29	81.37	86.73	126.01	101.70	–
*BnaC08g10640D*	23.17	24.83	40.29	85.99	32.39	37.60	Peptide transporter activity.
*BnaCnng57190D*	65.92	0.48	42.78	73.89	0.32	40.80	Nucleo-base trans-membrane transporter activity.
*BnaC06g15480D*	51.59	15.48	38.43	72.04	23.54	61.88	Peptide disulfide oxido-reductase activity; Secondary active trans-membrane transporter activity.
*BnaC04g50590D*	76.26	80.08	22.83	71.08	106.61	115.09	Water trans-membrane transporter activity.
*BnaC07g15960D*	24.44	16.93	22.68	69.25	24.68	10.34	Inorganic cation trans-membrane transporter activity.
*BnaA07g11370D*	98.66	20.51	157.57	68.50	19.53	188.85	Peroxidase activity; Amino acid trans-membrane transporter activity; iron ion binding.
*BnaAnng14550D*	30.79	28.59	35.98	67.78	31.70	15.74	Inorganic cation trans-membrane transporter activity.
*BnaA08g10860D*	110.30	59.24	87.63	66.84	49.77	92.90	Water trans-membrane transporter activity.

*Green-yellow color scale shows the values from highest to lowest*.

### Exploration of metal stress related molecular transporters

Similar to PKs, we also analyzed the metal toxicity-related MTs (Table 7). Data highlighted that main categories of metal transporters were related to amine transmembrane transporter activity, Cd and Ca channel activity, chloride channel activity, copper ion transmembrane transporter activity, hydrogen ion transmembrane transporter activity, iron, inorganic and ion transmembrane transporter activity, and manganese and metal ion trans-membrane. Besides, results also highlighted the MTs related to monovalent cation: hydrogen antiporter activity, nucleotide binding; copper ion transmembrane transporter activity, oxidoreductase activity; sugar: hydrogen symporter activity, peroxidase activity; amino acid trans-membrane transporter activity; iron ion binding, potassium ion symporter activity, protein transporter activity, sodium ion trans-membrane transporter activity and voltage-gated potassium channel activity in two cultivars under the three different treatments conditions.

**Table 7:** Represented the transcripts coding the molecular transporters (RPKM) related to metal stress in two cultivars of *B. napus* under the different treatment conditions i.e., Ck (control), 400 μM Cr and 400 μM Cr + 1 mM GSH.

**Gene ID**	**ZS 758**			**Zheda 622**			**GO Function**
	**Ck**	**Cr**	**Cr + GSH**	**Ck**	**Cr**	**Cr + GSH**	
*BnaC07g45360D*	86.24	31.76	39.48	50.51	25.06	15.87	Amine transmembrane transporter activity; peroxidase activity; iron ion binding
*BnaA04g07800D*	5.99	2.97	10.20	3.50	1.39	8.37	Amino acid transmembrane transporter activity; transition metal ion binding
**CD AND CA CHANNEL ACTIVITY, AND CATION BINDING**							
*BnaC08g49210D*	1.66	0.27	1.95	14.22	2.26	4.37	Cadmium and zinc ion trans-membrane transporter activity
*BnaA09g01640D*	8.20	5.76	9.94	7.73	7.60	10.54	Calcium channel activity
*BnaC09g00810D*	1.85	3.38	4.23	3.20	3.37	4.93	Calcium channel activity
*BnaC03g37860D*	2.83	8.50	3.92	4.02	9.77	4.97	Calcium ion transmembrane transporter activity; solute: cation antiporter activity; Cation and iron sulfur binding
*BnaC09g43660D*	4.49	8.15	3.77	5.06	7.82	4.15	Cation binding; oxidoreductase activity; iron-sulfur cluster binding
*BnaC02g04750D*	4.19	8.36	3.62	0.36	5.94	1.09	Cation binding; oxidoreductase activity; iron-sulfur cluster binding
*BnaC02g04740D*	0.67	4.97	1.31	0.06	3.24	0.30	Cation binding; oxidoreductase activity; iron-sulfur cluster binding
*BnaA02g21820D*	10.38	18.82	16.43	10.75	20.17	12.78	Cation-transporting ATPase activity; hydrogen ion transmembrane transporter activity
**CHLORIDE CHANNEL ACTIVITY**							
*BnaC07g24030D*	1.76	3.61	2.24	2.23	2.19	1.56	Chloride channel activity; hydrogen ion transmembrane transporter activity
*BnaC06g13300D*	5.46	7.55	2.77	5.44	7.68	3.10	Chloride channel activity; hydrogen ion transmembrane transporter activity
*BnaA07g15180D*	4.39	12.11	2.61	2.48	9.30	1.08	Chloride channel activity; hydrogen ion transmembrane transporter activity
**COPPER ION TRANSMEMBRANE TRANSPORTER ACTIVITY**							
*BnaC01g41080D*	0.00	19.21	0.00	0.13	9.32	0.00	Copper ion transmembrane transporter activity
*BnaCnng46010D*	0.00	12.54	0.00	0.00	6.98	0.00	Copper ion transmembrane transporter activity
*BnaA10g14850D*	40.11	69.37	74.01	56.25	83.58	82.56	Copper ion transmembrane transporter activity
*BnaC02g10130D*	11.23	2.39	7.33	1.75	1.24	2.11	Copper ion transmembrane transporter activity
*BnaC07g36020D*	9.08	8.39	3.03	13.85	12.86	0.66	Copper ion transmembrane transporter activity
*BnaA01g35020D*	2.54	54.13	0.70	2.87	37.22	0.33	Copper ion transmembrane transporter activity
*BnaA01g35030D*	2.54	164.02	1.96	6.04	200.23	1.87	Copper ion transmembrane transporter activity
*BnaC01g24930D*	1.40	47.52	1.16	8.03	32.77	2.21	Copper ion transmembrane transporter activity
*BnaA09g52720D*	7.54	6.74	9.49	11.04	7.51	10.16	Copper ion transmembrane transporter activity; identical protein binding
**HYDROGEN ION TRANSMEMBRANE TRANSPORTER ACTIVITY**							
*BnaC09g49950D*	18.54	45.96	26.44	22.63	46.08	28.16	Hydrogen ion transmembrane transporter activity
*BnaC07g38430D*	15.69	32.02	9.51	11.74	32.05	10.06	Hydrogen ion transmembrane transporter activity
*BnaA01g31400D*	14.95	12.91	20.51	15.38	13.49	15.36	Hydrogen ion transmembrane transporter activity
*BnaC05g11420D*	10.32	29.93	8.28	9.95	25.03	9.26	Hydrogen ion transmembrane transporter activity
*BnaA06g32380D*	1.97	3.88	1.12	2.63	3.33	0.66	Anion channel activity
*BnaAnng06000D*	5.47	16.94	7.55	4.49	6.51	2.72	ATPase activity, coupled to transmembrane movement of ions; nucleotide binding
*BnaC09g22670D*	184.36	2.67	100.21	166.56	2.36	86.79	Hydrogen ion transmembrane transporter activity; cation-transporting ATPase activity
*BnaC03g30820D*	7.48	14.91	11.96	7.52	13.00	9.94	Hydrogen ion transmembrane transporter activity; cation-transporting ATPase activity
*BnaC08g16740D*	16.82	7.79	23.44	16.92	10.80	15.18	Hydrogen ion transmembrane transporter activity; hydrolase activity
*BnaA02g01640D*	35.32	70.02	48.61	37.39	66.38	44.08	Hydrogen ion transmembrane transporter activity; transition metal ion binding
*BnaC03g05880D*	17.96	30.22	12.43	18.91	41.40	15.07	Hydrogen ion trans-membrane transporter activity; transition metal ion binding
*BnaC09g43620D*	16.35	22.41	16.52	17.50	23.93	14.79	Hydrogen ion trans-membrane transporter activity; transition metal ion binding
*BnaA07g11570D*	59.25	88.97	68.38	65.83	112.15	79.52	Hydrolase activity, acting on acid anhydrides, catalyzing trans-membrane movement of substances
*BnaC06g39330D*	9.49	19.05	9.19	10.16	16.21	9.05	Hydrolase activity, acting on acid anhydrides, catalyzing trans-membrane movement of substances
*BnaA07g35320D*	7.15	2.95	7.08	3.94	2.93	2.75	Iron ion transmembrane transporter activity; guanyl ribonucleotide binding
**IRON, INORGANIC AND ION TRANSMEMBRANE**							
*BnaA07g20060D*	3.14	2.29	5.08	4.27	2.03	4.50	Iron ion transmembrane transporter activity
*BnaA09g47380D*	4.00	5.25	10.66	34.07	13.17	27.40	Inorganic anion transmembrane transporter activity; CoA-ligase activity; binding
*BnaC08g41560D*	1.83	2.29	8.98	0.04	0.00	0.67	Inorganic anion transmembrane transporter activity; CoA-ligase activity; binding
*BnaA03g21880D*	3.80	4.96	4.74	3.50	5.64	5.07	Ion transmembrane transporter activity
*BnaCnng60870D*	0.08	1.19	2.49	0.00	0.09	0.12	Ion transmembrane transporter activity
*BnaA10g07330D*	2.80	0.11	5.68	3.54	0.10	5.30	Ion transmembrane transporter activity; protein binding; nucleotide binding
**MANGANESE AND METAL ION TRANSMEMBRANE**							
*BnaA04g23020D*	11.12	4.51	10.69	13.12	2.91	8.27	Manganese ion transmembrane transporter activity
*BnaC04g46360D*	9.67	3.40	9.90	11.29	4.69	7.81	Manganese ion transmembrane transporter activity
*BnaA05g34600D*	0.15	0.18	2.58	0.08	0.00	0.14	Manganese ion transmembrane transporter activity
*BnaA10g00220D*	13.21	2.04	9.98	12.28	2.21	9.54	Metal ion transmembrane transporter activity
*BnaA09g20220D*	4.07	0.04	2.48	1.62	0.04	1.30	Metal ion transmembrane transporter activity
*BnaAnng21960D*	3.13	7.65	6.46	6.70	8.53	6.94	Metal ion transmembrane transporter activity
*BnaA03g60370D*	3.00	7.40	4.03	4.22	11.30	5.10	Metal ion transmembrane transporter activity
*BnaA05g21170D*	2.99	0.37	5.53	3.82	0.11	2.84	Metal ion transmembrane transporter activity
*BnaC05g45460D*	2.73	2.20	2.48	1.52	1.28	1.70	Metal ion transmembrane transporter activity
*BnaA07g17260D*	2.67	2.32	3.90	3.90	2.52	6.09	Metal ion transmembrane transporter activity; hydro-lyase activity
*BnaA08g11580D*	10.91	0.87	9.35	6.86	1.43	5.47	Inorganic cation transmembrane transporter activity
**MISCELLANEOUS**							
*BnaC09g02990D*	6.00	6.79	11.29	10.13	7.60	15.36	Monovalent cation:hydrogen antiporter activity
*BnaC09g36680D*	3.93	0.17	1.36	1.47	0.10	1.00	Nucleotide binding; copper ion transmembrane transporter activity
*BnaCnng53190D*	10.93	16.37	20.11	17.63	18.06	20.86	Nucleotide binding; hydrogen ion trans-membrane transporter activity
*BnaA05g19250D*	3.53	6.27	7.50	7.03	8.82	7.97	Oxidoreductase activity; sugar: hydrogen symporter activity
*BnaC07g15280D*	315.81	29.43	239.51	42.97	13.27	188.46	Peroxidase activity; amino acid trans-membrane transporter activity; iron ion binding
*BnaA08g21730D*	247.75	31.17	112.01	24.03	6.58	141.00	Peroxidase activity; amino acid trans-membrane transporter activity; iron ion binding
*BnaA07g16500D*	0.00	0.47	0.00	0.00	0.15	0.00	Potassium ion symporter activity
*BnaA07g14320D*	194.99	9.90	232.21	117.99	13.01	140.71	Protein transporter activity
*BnaA02g36580D*	6.26	0.21	6.55	7.82	0.47	4.62	Sodium ion trans-membrane transporter activity
*BnaC05g32770D*	3.85	6.72	5.10	3.78	8.67	4.87	Sugar; hydrogen symporter activity
*BnaA03g13690D*	1.01	0.07	1.10	1.22	0.12	0.97	Voltage-gated potassium channel activity

### RT-PCR analysis of Cr and Cr + GSH responsive PKs and MTs

**Figure 8:**
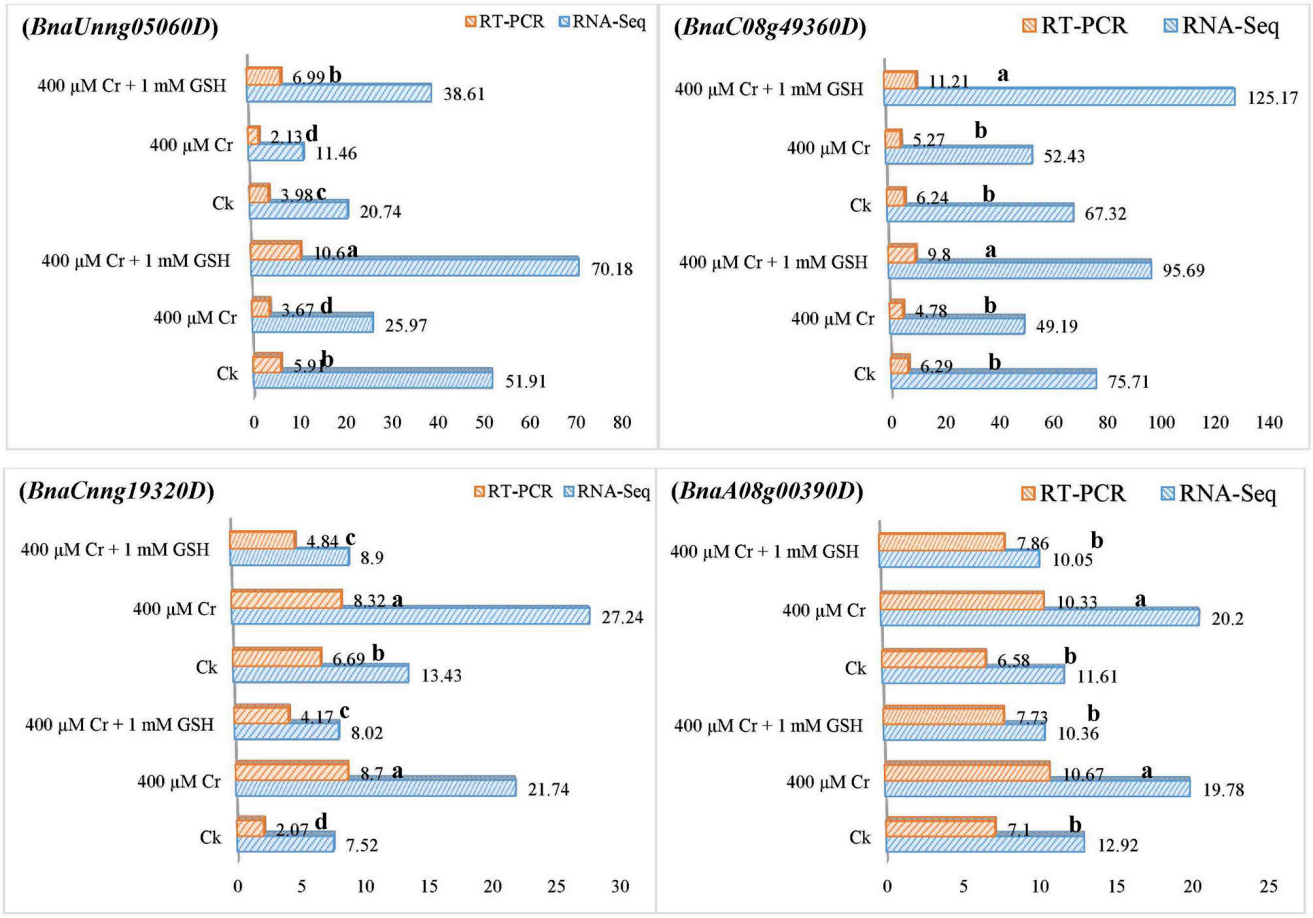
RNA-Seq and RT-PCR analyses of transcripts related to protein kinases expressed commonly in two cultivars i.e., ZS 758 and Zheda 622 of *Brassica napus*; *BnaUnng05060D* responsive to iron protein binding, single strand DNA binding, and protein kinase activity; *BnaC08g49360D* related to protein kinase activity, structural transducer activity and oxidoreductase activity; *BnaCnng19320D* coding the transition metal binding; and *BnaA08g00390D* responsice for the cation binding; phosphotransferase activity. Value of RNA-Seq adjusted as scale to 10. Lower case letters shows the significance level at 5% probability. Different letters indicate statistically significant differences among the treatments.

**Figure 9:**
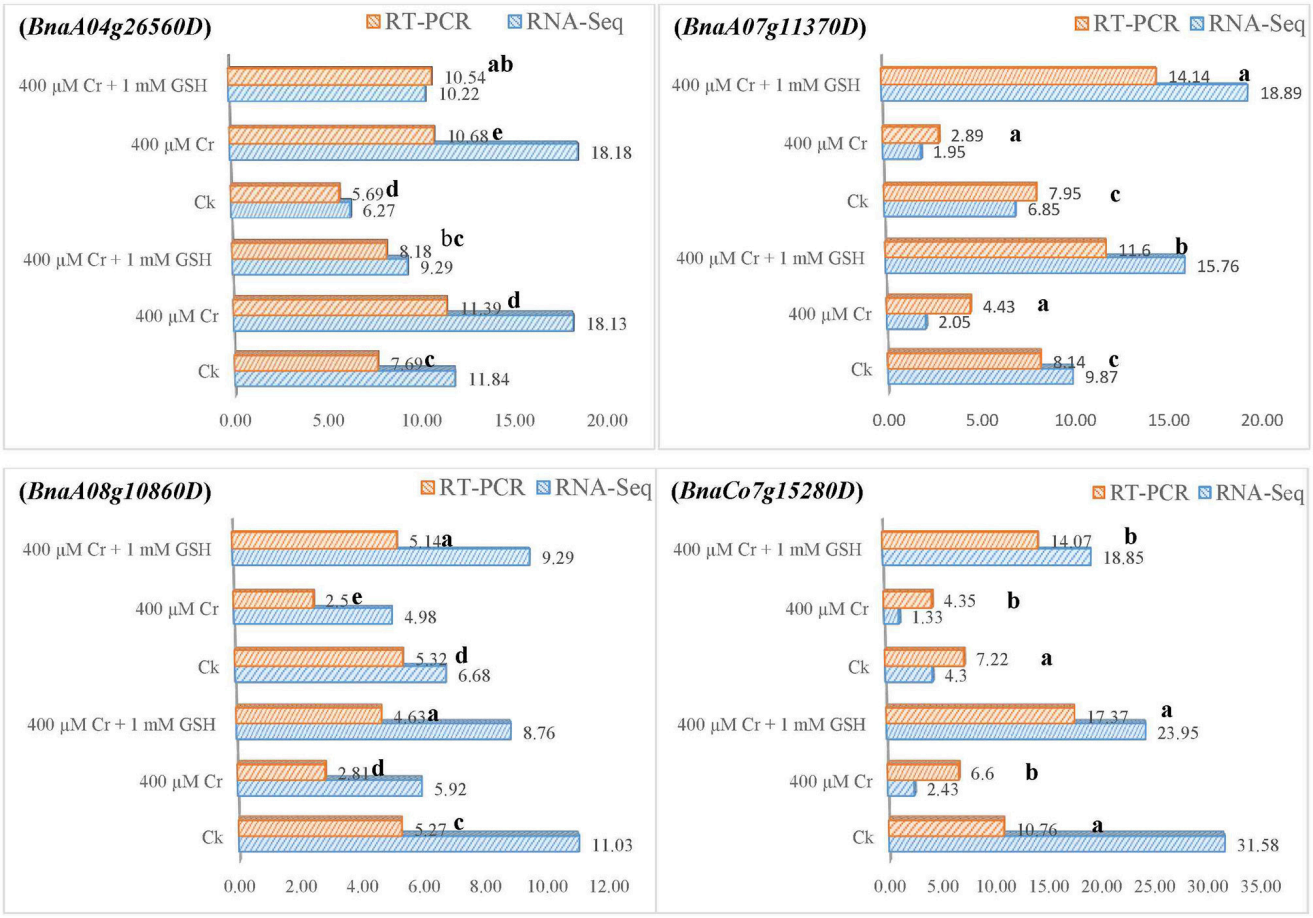
RNA-Seq and RT-PCR analyses of transcripts related to molecular transporters expressed commonly in two cultivars i.e., ZS 758 and Zheda 622 of *Brassica napus*; *BnaA04g26560D* coding for the water trans-membrane transporter activity; *BnaA07g11370D* related to Peroxidase activity; amino acid trans-membrane transporter activity; *BnaA08g10860D* corresponding to water trans-membrane transporter activity; and *BnaCo15280D* responsive for the Peroxidase activity; amino acid trans-membrane transporter activity. Value of RNA-Seq adjusted as scale to 10. Lower case letters shows the significance level at 5% probability. Different letters indicate statistically significant differences among the treatments.

In order to gain additional information, we further analyzed the RNA-Seq suggested transcripts that were categorized as PKs and MTs under Cr and Cr + GSH conditions. PKs results showed that *BnaCnng19320D* and *BnaAo00390D* transcripts were expressed higher under Cr alone vs. Ck, and Cr + GSH conditions. The fold change levels of these genes were increased to 4.20, 1.50, 1.24, and 1.57 than Ck in ZS 758 and Zheda 622, respectively (Figure 8). These transcripts were responsible for the transition metal and cation binding, and phospho-transferase activity. Similarly, in both cultivars, the *BnaUnng05060D* and *BnaCo49360D* transcripts were up-regulated under Cr + GSH combined conditions over Cr alone. These genes were up-regulated by 2.77-, 1.95-, 3.77-, and 2.39-fold than Cr alone in ZS 758 and Zheda 622, respectively. These transcripts were responsible for PKA, IPb, SSDNAb, STA, and ORA activities. In the case of MTs, *BnaAo26560D* was up-regulated more under Cr conditions in both cultivars. The increase was noticed by 1.48-fold in ZS 758 and 1.88-fold in Zheda 622 (Figure 9). This transcript encoded water trans-membrane transporter activity. Under Cr + GSH conditions, we found *BnaAo11370D, BnaAo10860D*, and *BnaCo15280D* transcripts showed higher expression than Cr alone condition. These genes were up-regulated as 2.62-, 1.65-, and 2.63-fold in ZS 758 and 4.89-, 2.06-, and 3.23-fold in Zheda 622. These transcripts were responsible for Fe binding in addition to peroxidase, amino acid trans-membrane transporter, and water trans-membrane transporter activities (Figure 9).

## Discussion

The response of *B. napus* to Cr-induced toxicity has been recently reported (Gill et al., 2014, 2015). However, there is no information regarding Cr-induced toxicity and its recovery after exogenously added GSH as studied by morpho-physiology, leaf mesophyll ultra-changes, kinome, and transportome analyses using both phenological parameters and the RNA-Seq method. It is evident from previous reports that GSH as a growth enhancer is useful under stress conditions, but the mechanisms behind growth regulation under stressful signals are not completely understood. The application of GSH as a growth enhancer under stress conditions has recently become the focus of several studies. Maybe it is due to that it acts as a primary plant's defender against the vast range of hazardous materials including abiotic and biotic elements. Being a phytochelin agent, GSH makes a complex with toxic elements before their induction prior to reach at an active/effective level (Flores-Cáceres et al., 2015). Furthermore, inside the plant cell, biosynthesis and accumulation of GSH can increase tolerant under the unfavorable environmental regime (Rausch et al., 2007; Foyer and Noctor, 2011). According to our knowledge, this is the first study that examined the influence of Cr and GSH in combination on *B. napus* cultivars for the mentioned above attributes.

The reduction in the plant growth-related attributes such as shoot and root lengths and fresh biomass are well-documented plant behaviors for studying heavy metal toxicity (Sharma and Dubey, 2005; Gill et al., 2015). In the current study, the root organ showed the most sensitivity to metal stress than stem and leaf organs. Results showed that stem and root length reductions were detected more in Zheda 622 than in any other cultivar. The suppression of root organs might be due to cell division reduction in roots caused by metal toxicity (Dey et al., 2009; Ali et al., 2013). Interestingly, we noticed that the percentage of root length deterioration was less than stem length deterioration in ZS 758. This is a clear indication that this cultivar has more tolerance/scavenging mechanisms against Cr-induced stress. A similar trend in Cr-induced stress was noticed in the case of fresh plant biomass attributes in both cultivars. The decrease in fresh plant biomass content might be the reason for inhibition of electron chain transport in the photosynthesis process (Mohanty et al., 1989). However, a beneficial role for GSH was noted under Cr stress conditions in all the above-mentioned growth-related parameters. Literature has evidenced that a non-enzymatic based antioxidant called GSH play a crucial role in scavenging the ROS, the photosynthesis by-products inside the cell organelles and also inactivates the singlet oxygen species with the help of ascorbate. Thus, GSH helps the plants to get recovery from negative effects of ROS on the plant's growth and development induced by hazardous environmental conditions (Mittler, 2002; Gill et al., 2016a). Moreover, GSH-ascorbate cycle is well-unspoken to detoxify the singlet oxygen species i.e. H_2_O_2_, OH^−^ and ${\text{O}}_{2}^{-.}$, in the result of this metal toxicity extent reduce to inactive level, hence plant can continue its normal growth (Ali et al., 2011). Besides, our investigation advocates that percentage of alleviation of Cr-induced stress was more in Zheda 622 than ZS 758. Based on these results, we can conclude that transportation/movement/growth regulator activities are required more when the pollution levels are higher.

A cellular ultrastructural study done by transmission electron microscopy (TEM) explained leaf ultrastructure and its resultant changes under different environmental conditions. Our study reported that Cr induced variations in *B. napus* leaf ultra-morphology, which was later restored after GSH treatment (Figure 1). Earlier studies have stated that Cr is mainly deposited in intercellular spaces, vacuoles, and cell walls, whereas little is found in chloroplasts, endoplasmic reticulum, and nuclei (Gill et al., 2015). However, even trace amounts of Cr can cause the significant changes in leaf ultrastructure (Islam et al., 2008). The TEM micrographs showed that under Cr-induced stress conditions alone, the size and number of starch grains had significantly increased. Breakage occurring in cell walls in addition to damaged thylakoid membranes and increased plastoglobuli structures were found in both cultivars (Figure 1). At the same time, when we applied GSH under Cr-induced stress conditions, the stability in *B. napus* leaf ultrastructure becomes apparent. Previously, also found well-developed chloroplast ultrastructure in barley with the application of exogenous GSH under cadmium toxicity (Wang et al., 2011). Improvement of leaf ultra-morphology with GSH under Cr-induced stress conditions may be due to GSH involvement in the up-regulation of defensive genes and stabilization of photosynthetic membranes (Ball et al., 2004; Carius et al., 2011). Furthermore, since GSH is a strong chelating agent and may possibly be a substrate for the S-transferase enzyme, it can form conjugates with toxic molecules. Hence, it helps cells to alleviate stress (Halliwell and Gutteridge, 2015). Thus, we can assume that GSH might be able to prevent metal element entry across the cell in order to protect the cell's ultrastructure.

A PK is an enzyme that regulates the several other proteins. It adds phosphate groups to a protein by a process known as phosphorylation. In this process, kinases enzymes change the functions of the target proteins by altering the enzyme activity, cellular location, or association with other proteins (Stone and Walker, 1995). In our study, genes that are related to PKA, ORA, IPb, SSDNAb, NAb, HA, metal ion binding, and phosphoenoylpyruvate carboxylase activities were found to be more prominent in both cultivars under Ck conditions. PKs such as SnRK2, CDPK, MAPK, GSK, and RPK are involved in the regulation of plant growth under several environmental conditions. These are hormone-mediated signaling (ABA-mediated) responses to osmotic stress, tissue injury, pathogenic damage, and metal-induced stress (Laurie and Halford, 2001). From previous literature studies, it is well-known that the SSDNAb protein plays a pivotal role in DNA replication regulation, recombination, and repair processes. Moreover, during DNA metabolism, this protein helps SSDNA to bind with several other proteins. In this process, it may act as a sliding platform that migrates on DNA via reptation (Kozlov et al., 2010; Zhou et al., 2011). Under Cr-induced toxicity, transcripts encoding NAb, PKA, IPB, STA, transition metal ion binding (TMIBP), and ORA had significantly increased in cultivar ZS 758. Among nuclear regulatory proteins, RNA-binding proteins are well-known mediators who control post-transcriptional RNA-metabolism during plant growth, its development stages, and stress responses (Lee and Kang, 2016). Previous studies have indicated that in human cells, cellular NAb proteins (Yasuda et al., 1995) positively control the process of DNA-transcription (Liu et al., 1998). Prominent NAb protein functions in the cell include regulation of the NA chaperone with the help of both SSDNA and RNAb activities (Armas et al., 2008). A well-known enzyme, nicotinamide adenine dinucleotide phosphate (NAD(P)H)-dependentQuinoneOxidoreductase-1 has been recognized as a protector against oxidative injury (Valderrama et al., 2006). Thus, up-regulation of genes regarding the NAb protein, kinases, and ORA has indicated that the cultivar ZS 758 demonstrates a more sophisticated and tolerant genome (Table 2).

On the other hand, TMIBP-related GO function was up-regulated in Zheda 622 under stress conditions (Table 3). The main role of this protein appears to interact selectively and non-covalently with any transition metal ion such as Fe, Cu, Mn, and Zn, all of which are essential minerals for healthy plant growth and development as long as their levels remain below the toxic threshold mark (Hennig, 1986; Babor et al., 2008). Thus, we can conclude that due to increased toxic metal ions such as Cr, the TMIBP expression level also increased as a mechanism to cope with the adverse impacts of Cr-induced stress. Hence, these proteins play a crucial role in scavenging the metal ion(s) and also play an important role in several biological processes. Since we added exogenous GSH to the Cr solution, it not only had further improved enzyme activities as described above but also increased the expression of the hydrolase-encoding gene. It has been well-documented that GSH (as a low molecular weight thiol) plays a major role in scavenging metal ions, thus preventing the toxicity-induced adverse effects on plant's organs (Rouhier et al., 2008; Foyer and Noctor, 2011). Moreover, GSH acts as a dual protective shield in plants against both abiotic and biotic stress. Due to its unique structure, it can attain a particular position in between the plant defensors (cellular reductants) and reactive oxygen species; hence, the GSH system works better for signaling functions (Noctor et al., 2012). Our results suggest that cultivar Zheda 622 was more responsive under GSH + Cr conditions. Thus, the recent behavior of Zheda 622 indicates that the active involvement of GSH as a growth enhancer appears to be greater in more susceptible genotypes.

Similar to protein kinases, we also explored MTs in two cultivars under different treatment conditions. The phenomenon of cellular molecular transport involves elemental ion or material transport through the plasma or cell membrane. Furthermore, it takes place via passive and active processes. In passive transport no energy required, but in active transport, ATP molecules are needed (Arcizet et al., 2008). Thus, for both processes, there are specialized transporters that play a significant role to either block the toxic metal ion or permit more H_2_O molecules to move across the cell membranes from tissue to tissue and then organ to organ. Similarly, in our study, the peroxidase, AA transmembrane transporter, and iron binding-related activities were more prominent in ZS 758 (Table 4). Earlier work has confirmed that ascorbate peroxidase (APX) enzymes play a fundamental role in the ascorbate-glutathione cycle, in which APX enzyme detoxifies H_2_O_2_ by converting it into H_2_O; hence, it helps plants to alleviate stress (Caverzan et al., 2012). The role of AA transporters has also been well-documented. These transporters play a crucial role in plant growth and development by providing logistic support (Ortiz-Lopez et al., 2000). Likewise, Fe binding proteins maintain its cellular content level; Fe is required for DNA synthesis, respiration, chlorophyll synthesis, and to stabilization of both chloroplast structure and function. Thus, this protein protects plants from a nutritional disorder that can result in low yield and poor quality (Schmidt, 1993).

On the other hand, hydrogen ion transmembrane transporter (HITT), cation-transporting ATPase, and protein transporter activities were more apparent in Zheda 622 than in ZS 758 in Cks (Table 5). It has been shown that the HITTA protein enables the transfer of hydrogen ions from one side of a membrane to the other, which has been termed proton transporter activity (PTA) (Morsomme and Boutry, 2000). Under Cr treatment, expression of two genes was significantly higher than in control conditions. One was characterized as a novel gene (have no GO function) and the second was related to water transmembrane transporter activity. Earlier reports have stated that the expression of a well-known protein (aquaporin), which facilitates water movement across the membrane under unfavorable conditions (Luu et al., 2007), demonstrated increased expression; thus, it appears the increase in expression of water transport-related proteins in this study alleviated the adverse effects of metals on *B. napus* plants. The application of exogenous GSH provided stress relief in two cultivars. Moreover, GSH indicated which transcripts related to PTA; under stress conditions, secondary active transmembrane and organic acid transmembrane transporter activities expressed at even higher levels than the control in both cultivars. Interestingly, the overall role of GSH was more apparent in ZS 758. Based on these results, we can conclude that GSH plays a pivotal role in activation of the genes related to molecular transporters, which are mostly present in root, and stem organs of a plant (specifically cultivar ZS 758 in this study) and provides a better transport system that can prevent the transport of hazardous metal ions. In this way, the plants, with the help of exogenous regulators such as GSH, can create a more powerful protective shield against the toxic pollutants.

## Conclusion

Findings from the present study indicated that changes in growth-related attributes, cellular structural alterations, PKs, and MTs occurred when plant cells were exposed to the Cr and GSH interactive environment. In this study, Cr significantly affected the cultivar Zheda 622 as revealed by fresh plant biomass. The TEM micrographs proved that cultivar Zheda 622 was severely damaged under Cr stress. Data regarding the PKs provided specific cultivar information about metal stress. For instance, in cultivar ZS 758, gene expression of NAb proteins that controlled post-transcriptional RNA-metabolism during plant growth was up-regulated. Similarly, under Cr stress conditions, cultivar Zheda 622 showed higher expression of genes that were responsive to TMIBP, which interacted with both selectively and non-covalently with transition metals that are essential minerals for healthy plant growth and development. Moreover, transcripts encoding the peroxidase enzymes that convert H_2_O_2_ into H_2_O were significantly increased in cultivar ZS 758 than in other cultivars. Exogenously applied GSH successfully alleviated the adverse effects of Cr in *B. napus* seedlings; it helped to improve plant growth, cell ultrastructure, PKs, and MTs. In order to examine the precise and accurate ameliorative role of GSH under Cr stress conditions in future studies, a combination of soil environment and RNA-Seq data is needed.

## Availability of RNA-Seq data

The transcriptome induced raw data have been submitted to the National Centre for Biotechnology Information (NCBI), the Sequence Read Archive (SRA) database and could access with the accession number of “SAMN04455792.”

## Author contributions

RG, BA, SL, and WZ designed the experiments; RG, MG, and SY performed the experiments; RG, CT, FI, TM, and SA analyzed the data; RG, BA, BM, SL, and WZ wrote the paper.

### Conflict of interest statement

The authors declare that the research was conducted in the absence of any commercial or financial relationships that could be construed as a potential conflict of interest.
