# Supplementary material for: Reduced Glutathione Mediates Pheno-Ultrastructure, Kinome and Transportome in Chromium-Induced Brassica napus L
Source: Front Plant Sci. 2017 Dec 11;8:2037. doi: 10.3389/fpls.2017.02037 (PMC5732361; doi:10.3389/fpls.2017.02037)
Supplement: Supplementary file 1 [file Table1.DOC]

**Table S1** Oligonucleotides sequences used for the elite transcripts related to protein kinases and molecular transporters in the qRT-

**PCR analysis.**

| **Gene ID** | **Forward** | **Reverse** | **Gene Description** |
| --- | --- | --- | --- |
| ***Protein Kinases*** | | | |
| BnaUnng05060D | GAAACGGAACGGTCTCAC | GAGGATACGGTGGAGGTG | Iron protein binding, single strand DNA binding, protein kinase activity (PKA), structural transducer activity (STA) and oxido-reductase activity (ORA) |
| BnaC08g49360D | AACATTGCGTTTCCAGAT | TCCGTACTTCCGTTTCTG | PKA, STA and ORA |
| BnaCnng19320D | AAAGGGATGGTTTGAGCC | AGCAATTCGAGCAGATGG | Transition metal binding |
| BnaA08g00390D | TCATAAACAACAACCGAAAC | TTGACATTGGGAGGAACA | Cation binding; phosphotransferase activity |
| ***Molecular Transporters*** | | | |
| BnaA04g26560D | GTCACCACCACCTTCCAA | AGTTCCTATGTTTCATTTCTTA | Water trans-membrane transporter activity |
| BnaA07g11370D | GGAGGAGCCAATCACAGC | AGAACCAACCGACCAACA | Peroxidase activity; amino acid trans-membrane transporter activity |
| BnaA08g10860D | TCTCCGCTACTGATGCTA | GAAGATAACGGCTGCTCC | Water trans-membrane transporter activity |
| BnaCo7g15280D | TTTCGGATTGTGAGTGAA | GGGACGGTACTGCAAGAA | Peroxidase activity; amino acid trans-membrane transporter activity |
| Actin | TTGGGATGGACCAGAAGG | TCAGGAGCAATACGGAGC | Standard |
