# Supplementary material for: Reduced Glutathione Mediates Pheno-Ultrastructure, Kinome and Transportome in Chromium-Induced Brassica napus L
Source: Front Plant Sci. 2017 Dec 11;8:2037. doi: 10.3389/fpls.2017.02037 (PMC5732361; doi:10.3389/fpls.2017.02037)
Supplement: Supplementary file 2 [file Table2.DOC]

**Table S2** Gene, length (bps) and coverage (%) data of protein kinases under the different treatment

conditions i.e. Ck (control), 400 µM Cr and 400 µM Cr + 1 mM GSH while ZS 758 under Ck takes as a standard.

| **Gene ID** | **Length (bps)** | | **ZS 758** | | | **Zheda 622** | | |
| --- | --- | --- | --- | --- | --- | --- | --- | --- |
|  |  |  | **Ck** | **Cr** | **Cr + GSH** | **Ck** | **Cr** | **Cr + GSH** |
| BnaA09g52790D | 2719 | | 38.95% | 32.22% | 36.89% | 32.36% | 30.12% | 36.67% |
| BnaC08g49360D | 1072 | | 85.91% | 88.62% | 85.07% | 84.89% | 86.66% | 88.06% |
| BnaC01g00280D | 1070 | | 91.40% | 91.31% | 91.68% | 62.71% | 90.28% | 89.91% |
| BnaAnng35580D | 1016 | | 98.03% | 98.43% | 98.52% | 78.25% | 76.57% | 95.77% |
| BnaUnng05060D | 1115 | | 91.21% | 77.85% | 91.84% | 70.58% | 61.35% | 75.52% |
| BnaCnng69940D | 1825 | | 87.23% | 79.34% | 86.36% | 79.23% | 77.37% | 88.66% |
| BnaA08g16610D | 1043 | | 93.48% | 97.22% | 96.26% | 81.69% | 80.82% | 91.75% |
| BnaA01g05410D | 1053 | | 90.31% | 91.26% | 88.41% | 71.51% | 85.47% | 69.23% |
| BnaA09g54020D | 2249 | | 68.74% | 14.58% | 66.83% | 68.92% | 18.67% | 63.18% |
| BnaCnng22330D | 524 | | 32.63% | 26.53% | 40.84% | 31.68% | 25.57% | 39.69% |
| BnaA01g30320D | | 2459 | 73.65% | 30.95% | 67.91% | 68% | 47.30% | 67.34% |
| BnaC03g45180D | 2261 | | 67.40% | 14.77% | 57.72% | 67.27% | 15.44% | 67.05% |
| BnaC01g38270D | 2482 | | 73.05% | 35.09% | 61.24% | 66.04% | 35.50% | 66.48% |
| BnaA08g02530D | 2182 | | 81.90% | 57.93% | 74.52% | 86.30% | 64.71% | 75.34% |
| BnaA04g12350D | 1101 | | 72.39% | 70.94% | 80.20% | 71.66% | 68.57% | 76.02% |
| BnaA09g26590D | 2975 | | 59.23% | 44.03% | 50.99% | 48.67% | 36.13% | 53.55% |
| BnaCnng53320D | 2731 | | 27.24% | 23.69% | 25.19% | 24.97% | 21.86% | 27.39% |
| BnaC03g60490D | 2090 | | 51.63% | 47.42% | 51% | 53.64% | 55.60% | 54.21% |
| BnaC04g48440D | 5397 | | 73.08% | 55.23% | 56.81% | 63.94% | 50.71% | 59.31% |
| BnaA09g00250D | 2360 | | 77.25% | 17.20% | 70.89% | 67.67% | 20.72% | 68.26% |

Note: Green-white-red color scale shows the values from the highest level to lowest.
