# Supplementary material for: Reduced Glutathione Mediates Pheno-Ultrastructure, Kinome and Transportome in Chromium-Induced Brassica napus L
Source: Front Plant Sci. 2017 Dec 11;8:2037. doi: 10.3389/fpls.2017.02037 (PMC5732361; doi:10.3389/fpls.2017.02037)
Supplement: Supplementary file 3 [file Table3.DOC]

**Table S3** Gene length (bps) and coverage (%) data of protein kinases under the different

treatment conditions i.e. Ck (control), 400 µM Cr and 400 µM Cr + 1 mM GSH while Zheda 622 under Ck takes as a standard.

| **Gene ID** | **Length (bps)** | **ZS 758** | | | **Zheda 622** | | |
| --- | --- | --- | --- | --- | --- | --- | --- |
|  |  | **CK** | **Cr** | **Cr + GSH** | **CK** | **Cr** | **Cr + GSH** |
| BnaC08g49360D | 1072 | 85.91% | 88.62% | 85.07% | 84.89% | 86.66% | 88.06% |
| BnaA08g16610D | 1043 | 93.48% | 97.22% | 96.26% | 81.69% | 80.82% | 91.75% |
| BnaA10g05680D | 3302 | 0.00 | 4.82% | 52.70% | 77.86% | 33.40% | 72.23% |
| BnaA01g05410D | 1053 | 90.31% | 91.26% | 88.41% | 71.51% | 85.47% | 69.23% |
| BnaA08g02530D | 2182 | 81.90% | 57.93% | 74.52% | 86.30% | 64.71% | 75.34% |
| BnaC01g00280D | 1070 | 91.40% | 91.31% | 91.68% | 62.71% | 90.28% | 89.91% |
| BnaC03g60490D | 2090 | 51.63% | 47.42% | 51% | 53.64% | 55.60% | 54.21% |
| BnaC03g45180D | 2261 | 67.40% | 14.77% | 57.72% | 67.27% | 15.44% | 67.05% |
| BnaUnng05060D | 1115 | 91.21% | 77.85% | 91.84% | 70.58% | 61.35% | 75.52% |
| BnaA09g54020D | 2249 | 68.74% | 14.58% | 66.83% | 68.92% | 18.67% | 63.18% |
| BnaAnng35580D | 1016 | 98.03% | 98.43% | 98.52% | 78.25% | 76.57% | 95.77% |
| BnaC09g29780D | 3533 | 58.53% | 57.60% | 71.55% | 74.87% | 58.90% | 65.78% |
| BnaCnng19320D | 2054 | 73.47% | 81.16% | 77.07% | 80.82% | 84.32% | 81.16% |
| BnaA01g30320D | 2459 | 73.65% | 30.95% | 67.91% | 68% | 47.30% | 67.34% |
| BnaA08g00390D | 1132 | 62.54% | 69.08% | 61.66% | 58.39% | 68.46% | 64.31% |
| BnaA06g01430D | 2613 | 63.30% | 26.22% | 73.40% | 80.37% | 27.44% | 72.25% |
| BnaC04g48440D | 5397 | 73.08% | 55.23% | 56.81% | 63.94% | 50.71% | 59.31% |
| BnaC01g38270D | 2482 | 73.05% | 35.09% | 61.24% | 66.04% | 35.50% | 66.48% |
| BnaA05g16480D | 1884 | 52.28% | 2.60% | 52.34% | 51.06% | 7.80% | 51.17% |
| BnaC03g37820D | 2740 | 59.05% | 40.18% | 58.07% | 62.23% | 40.66% | 64.56% |

Note: Green-yellow color scale shows the values from the highest level to lowest.
