# Supplementary material for: Reduced Glutathione Mediates Pheno-Ultrastructure, Kinome and Transportome in Chromium-Induced Brassica napus L
Source: Front Plant Sci. 2017 Dec 11;8:2037. doi: 10.3389/fpls.2017.02037 (PMC5732361; doi:10.3389/fpls.2017.02037)
Supplement: Supplementary file 4 [file Table4.DOC]

**Table S4** Gene length (bps) and coverage (%) data of transporters under the different treatment

conditions i.e. Ck (control), 400 µM Cr and 400 µM Cr + 1 mM GSH while ZS 758 under Cks take as a standard.

| **Gene ID** | **Length (bps)** | **ZS 758** | | | **Zheda 622** | | |
| --- | --- | --- | --- | --- | --- | --- | --- |
|  |  | **Ck** | **Cr** | **Cr + GSH** | **Ck** | **Cr** | **Cr + GSH** |
| BnaC07g15280D | 3918 | 78.25% | 43.87% | 72.13% | 52.68% | 40.86% | 64.80% |
| BnaC03g29960D | 1581 | 90.51% | 27.58% | 88.24% | 84% | 35.29% | 82.92% |
| BnaA08g21730D | 3563 | 56.30% | 45.86% | 47.63% | 41.06% | 35.36% | 62.25% |
| BnaCnng66500D | 1046 | 63.67% | 19.79% | 61.28% | 61.95% | 34.13% | 63.96% |
| BnaC08g19360D | 3579 | 71.25% | 51.77% | 56.02% | 54.18% | 45.60% | 64.82% |
| BnaA03g25540D | 1585 | 89.65% | 15.77% | 87.57% | 87.76% | 45.05% | 86.56% |
| BnaA07g14320D | 1249 | 90.95% | 44.52% | 84.47% | 75.02% | 48.20% | 82.79% |
| BnaC09g22670D | 1666 | 89.92% | 31.75% | 86.31% | 87.33% | 39.08% | 84.15% |
| BnaA09g20320D | 1522 | 83.11% | 37.25% | 80.81% | 82.72% | 37.25% | 80.95% |
| BnaA04g26560D | 1740 | 72.01% | 74.25% | 71.44% | 66.15% | 80.75% | 76.32% |
| BnaA02g28130D | 1723 | 84.21% | 82.94% | 68.43% | 76.03% | 86.24% | 61.98% |
| BnaC04g40040D | 3074 | 58.82% | 48.08% | 58.46% | 55.53% | 52.54% | 55.95% |
| BnaA08g10860D | 1653 | 83.61% | 79.19% | 81.67% | 81.61% | 78.10% | 83.97% |
| BnaC02g36210D | 1760 | 88.12% | 70.74% | 79.49% | 78.81% | 73.69% | 82.56% |
| BnaC09g25660D | 842 | 78.15% | 31.71% | 66.86% | 76.72% | 44.30% | 73.40% |
| BnaA07g11370D | 6281 | 35.06% | 25.84% | 33.96% | 32.96% | 26.73% | 36.17% |
| BnaA07g16540D | 823 | 82.75% | 69.14% | 68.53% | 69.02% | 66.34% | 77.04% |
| BnaA03g24090D | 705 | 47.38% | 6.95% | 45.67% | 47.23% | 25.96% | 46.52% |
| BnaA02g21070D | 5290 | 18% | 16.52% | 19.26% | 17.16% | 17.11% | 19.55% |
| BnaC07g45360D | 2737 | 51.26% | 48.15% | 49.32% | 50.53% | 49.76% | 48.48% |

Note: Green-white color scale shows the values from the highest to lowest.
