# Supplementary material for: Reduced Glutathione Mediates Pheno-Ultrastructure, Kinome and Transportome in Chromium-Induced Brassica napus L
Source: Front Plant Sci. 2017 Dec 11;8:2037. doi: 10.3389/fpls.2017.02037 (PMC5732361; doi:10.3389/fpls.2017.02037)
Supplement: Supplementary file 5 [file Table5.DOC]

**Table S5** Gene length (bps) and coverage (%) data of transporters under the different treatment

conditions i.e. Ck (control), 400µM Cr and 400 µM Cr + 1 mM GSH while Zheda 622 under Ck takes as astandard.

| **Gene ID** | **Length (bps)** | **ZS 758** | | | **Zheda 622** | | |
| --- | --- | --- | --- | --- | --- | --- | --- |
|  |  | **Ck** | **Cr** | **Cr + GSH** | **Ck** | **Cr** | **Cr + GSH** |
| BnaA03g25540D | 1585 | 89.65% | 15.77% | 87.57% | 87.76% | 45.05% | 86.56% |
| BnaA09g20320D | 1522 | 83.11% | 37.25% | 80.81% | 82.72% | 37.25% | 80.95% |
| BnaC09g22670D | 1666 | 89.92% | 31.75% | 86.31% | 87.33% | 39.08% | 84.15% |
| BnaC03g29960D | 1581 | 90.51% | 27.58% | 88.24% | 84% | 35.29% | 82.92% |
| BnaC01g29930D | 1881 | 51.94% | 50.45% | 84.69% | 81.92% | 51.52% | 88.57% |
| BnaCnng66500D | 1046 | 63.67% | 19.79% | 61.28% | 61.95% | 34.13% | 63.96% |
| BnaA08g05990D | 3120 | 66.67% | 61.57% | 68.40% | 65.29% | 65.38% | 65.90% |
| BnaA07g14320D | 1249 | 90.95% | 44.52% | 84.47% | 75.02% | 48.20% | 82.79% |
| BnaC04g40040D | 3074 | 58.82% | 48.08% | 58.46% | 55.53% | 52.54% | 55.95% |
| BnaA06g13930D | 1527 | 93.12% | 36.80% | 86.90% | 86.51% | 51.08% | 89.19% |
| BnaA07g16540D | 823 | 82.75% | 69.14% | 68.53% | 69.02% | 66.34% | 77.04% |
| BnaA02g01980D | 2070 | 68.45% | 69.66% | 70.63% | 70.82% | 72.42% | 66.96% |
| BnaC08g10640D | 3042 | 51.91% | 57.30% | 65.09% | 63.64% | 59.04% | 58.09% |
| BnaCnng57190D | 1612 | 87.41% | 6.70% | 88.71% | 88.59% | 9.12% | 82.32% |
| BnaC06g15480D | 923 | 72.81% | 69.34% | 72.91% | 85.59% | 66.09% | 76.60% |
| BnaC04g50590D | 1821 | 70.29% | 66.72% | 57.39% | 62.49% | 82.70% | 66.89% |
| BnaC07g15960D | 4518 | 62.37% | 53.43% | 56.46% | 62.26% | 49.29% | 46.66% |
| BnaA07g11370D | 6281 | 35.06% | 25.84% | 33.96% | 32.96% | 26.73% | 36.17% |
| BnaAnng14550D | 2439 | 74.70% | 73.64% | 76.10% | 69.58% | 65.52% | 59.94% |
| BnaA08g10860D | 1653 | 83.61% | 79.19% | 81.67% | 81.61% | 78.10% | 83.97% |

Note: Green-yellow color scale shows the values from the highest to lowest.
