# Supplementary figures and images for: Reduced Glutathione Mediates Pheno-Ultrastructure, Kinome and Transportome in Chromium-Induced Brassica napus L
Source: Front Plant Sci. 2017 Dec 11;8:2037. doi: 10.3389/fpls.2017.02037 (PMC5732361; doi:10.3389/fpls.2017.02037)

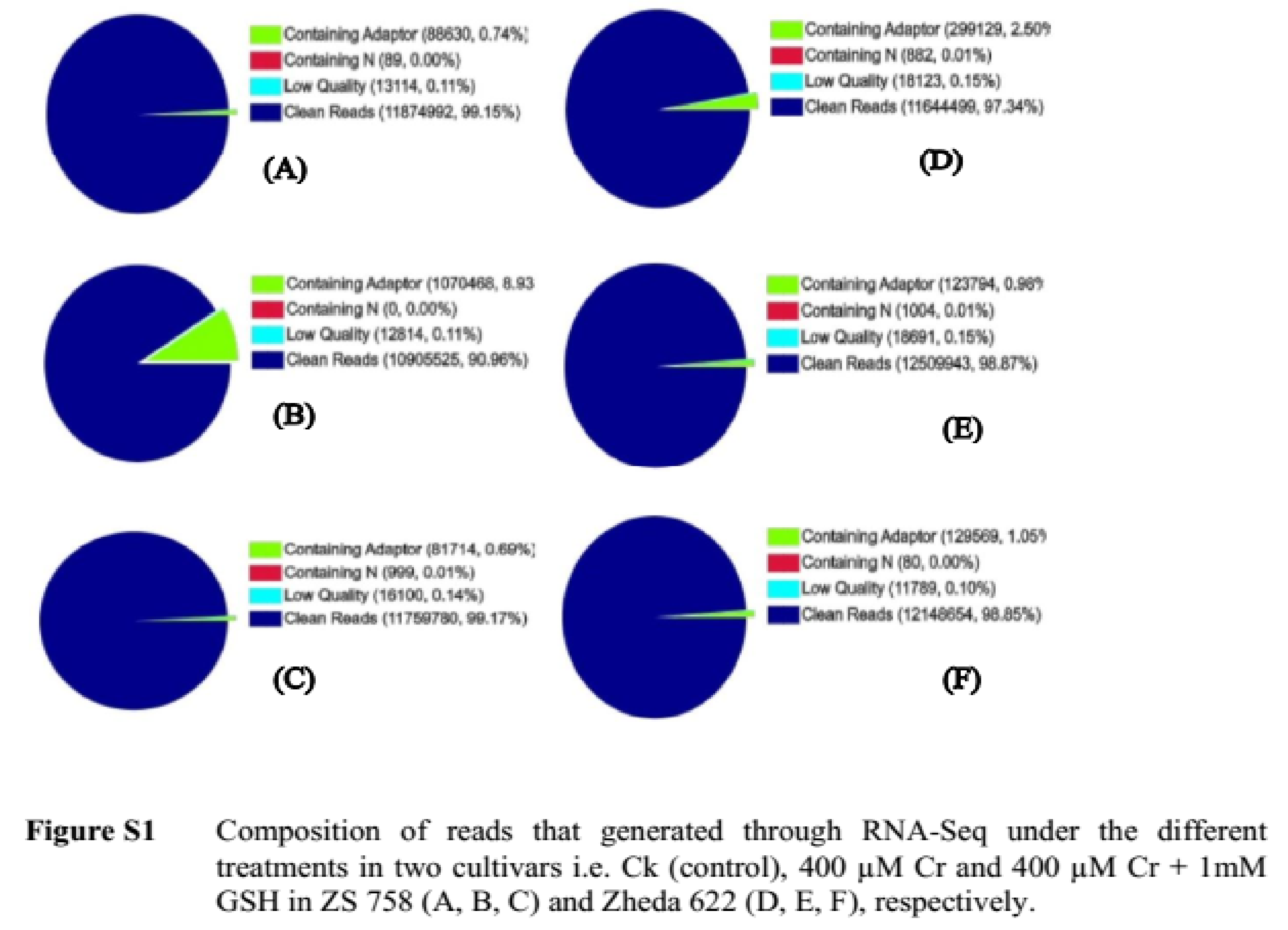

Supplement: Supplementary file 6 [file Image1.TIF]
